# Reprogramming of miR-181a/DNA methylation patterns contribute to the maternal nicotine exposure-induced fetal programming of cardiac ischemia-sensitive phenotype in postnatal life

**DOI:** 10.7150/thno.48297

**Published:** 2020-09-26

**Authors:** Jie Jian, Peng Zhang, Yong Li, Bailin Liu, Yanyan Zhang, Lubo Zhang, Xuesi M Shao, Jian Zhuang, Daliao Xiao

**Affiliations:** 1 Lawrence D. Longo, MD Center for Perinatal Biology, Department of Basic Sciences, Loma Linda University School of Medicine, Loma Linda, California, USA.; 2 Department of Cardiac Surgery, Guangdong Cardiovascular Institute, Guangdong Provincial People's Hospital, Guangdong Academy of Medical Sciences, Guangzhou, China.; 3 Department of Neurobiology, David Geffen School of Medicine at UCLA, University of California at Los Angeles, Los Angeles, California, USA.

**Keywords:** Perinatal nicotine, cardiac ischemia/reperfusion injury, miR-181a, DNA methylation

## Abstract

**Background:** E-cigarette and other novel electronic nicotine delivery systems (ENDS) have recently entered the market at a rapid pace. The community desperately needs answers about the health effects of ENDS. The present study tested the hypothesis that perinatal nicotine exposure (PNE) causes a gender-dependent increase in vulnerability of the heart to ischemia-reperfusion (I/R) injury and cardiac dysfunction in male rat offspring *via* reprogramming of the miRNA-181a (miR-181a)-mediated signaling pathway and that miR-181a antisense could rescue this phenotype.

**Methods:** Nicotine or saline was administered to pregnant rats *via* subcutaneous osmotic minipumps from gestational day 4 until postnatal day 10. Cardiac function and molecular biological experiments were conducted in ~3- month-old offspring.

**Results:** PNE enhanced I/R-induced cardiac dysfunction and infarction in adult male but not in female offspring, which was associated with miR-181a over-expression in left ventricle tissues. In addition, PNE enhanced offspring cardiac angiotensin receptor (ATR) expressions *via* specific CpG hypomethylation of AT_1_R/AT_2_R promoter. Furthermore, PNE attenuated cardiac lncRNA H19 levels, but up-regulated cardiac TGF-β/Smads family proteins and consequently up-regulated autophagy-related protein (Atg-5, beclin-1, LC3 II, p62) expression in the male offspring. Of importance, treatment with miR-181a antisense eliminated the PNE's effect on miR-181a expression/H19 levels and reversed PNE-mediated I/R-induced cardiac infarction and dysfunction in male offspring. Furthermore, miR-181a antisense also attenuated the effect of PNE on AT_1_R/AT_2_R/TGF-β/Smads/autophagy-related biomarkers in the male offspring.

**Conclusion:** Our data suggest that PNE could induce a reprogramming of cardiac miR-181a expression/DNA methylation pattern, which epigenetically modulates ATR/TGF-β/autophagy signaling pathways, leading to gender-dependent development of ischemia-sensitive phenotype in postnatal life. Furthermore, miR-181a could severe as a potential therapeutic target for rescuing this phenotype.

## Introduction

It is well recognized that tobacco smoking is one of the major risk factors of cardiovascular disease. Of the most concern, as rates of combustible cigarette use fell to historic lows, the tobacco industry has introduced e-cigarettes and other novel electronic nicotine delivery systems (ENDS) to addict a new generation of customers to nicotine products 1. However, there is insufficient data and scientific evidence in understanding of the long-term effects of nicotine addiction in pregnant women, children and youth. Although substantial evidence showed that maternal tobacco smoking is positively and robustly associated with cardiovascular disease in offspring 2, the underlying molecular mechanisms remain largely elusive.

MicroRNA (miRNA) is a member of small noncoding RNA family with a single strand of 18-25 nucleotides regulating expression of multiple target genes at the post-transcriptional level 3. A growing body of evidence suggests the importance of miRNAs in epigenetic inheritance and aberrant development of cardiovascular disease 4, 5. Recent studies in animal models and humans have shown that levels of miRNA-181a in the heart and blood are significantly increased in chronic heart failure, infarction fibrosis, hypertrophy and myocardial infarction patients 6-9. Furthermore, therapeutic downregulation of miR-181a resulted in the attenuation of myocardial fibrosis and hypertrophy, rescuing the damaged heart after myocardial infarction 8. These studies suggest that miR-181a could be one of the potential novel biomarkers for myocardial infarction. Our previous studies also revealed PNE selectively enhanced vascular miR181a levels, which directly regulated coronary vascular tone in offspring 10. Furthermore, our previous studies have demonstrated that maternal nicotine exposure enhances heart susceptibility to I/R injury and cardiac dysfunction *ex vivo* in offspring 11. However, it is unknown whether the changes of miRNAs play a role in perinatal nicotine-mediated exaggerated heart I/R injury and cardiac dysfunction. On the other hand, the long noncoding RNA (lncRNA) H19 is a highly abundant and conserved imprinted gene, which has been implicated in many essential biological processes and cardiovascular diseases 12. However, the functional role of lncRNA H19 in the heart and its relationship with miR181a remains unknown. In addition to miRNAs, DNA methylation is another one of the key epigenetic mechanisms which can directly regulate target gene expression without a change of the gene sequence. Fetal exposure to adverse stresses could influence the global and specific DNA methylation profiles late in life. Recent studies have shown that nicotine exposure alters cardiac gene expression *via* changes in DNA methylation pattern, which contribute to the etiology of smoking-associated cardiac defects 13. These studies lead to a hypothesis that aberrant miR-181a/DNA methylation patterns are the potential epigenetic molecular link between PNE and development of cardiovascular dysfunction in postnatal life.

Changes of miR-181a/DNA methylation could alter the expression of downstream target genes. Among these target genes, neuro-hormonal factors, such as angiotensin II (Ang II) and transforming growth factor (TGF) have been proven to regulate cardiac remodeling 14 and trigger cardiovascular disease 15. Ang II plays a crucial role in regulation of cardiac hypertrophy *via* activation of angiotensin II receptor type 1 (AT_1_R) and type 2 (AT_2_R). Alteration of AT_1_R and AT_2_R expression has been shown in cardiovascular dysfunction and cardiac remodeling 16. In addition, transforming growth factor beta (TGF-β) is a pleiotropic cytokine belonging to the transforming growth factor superfamily. Activation of TGF-β receptor I (TβRI) *via* TGF-β propagates downstream intracellular signals through the Smad proteins family 17. TGF-β is a crucial regulator of fibrogenic heart disease. Over-expression of TGF-β1 in mice model shows a significant left ventricular hypertrophy along with interstitial fibrosis 18. The fibrogenic and hypertrophic actions of endogenous TGF-β have been shown to contribute to the pathogenesis of cardiomyopathic conditions 19. In recent years, autophagy has been demonstrated as a major regulator of cardiac homeostasis. Whereas, in pathological cases aberrant autophagy appears to facilitate cell death and morbidity, excess of autophagy or autophagy deficiency could damage cell organelles and consequently induce tissue/organ dysfunction 20.

In the present study, we first developed a nicotine-exposed pregnant rat model to examine whether PNE caused a gender-dependent increase in vulnerability of the heart to ischemia-reperfusion (I/R) injury and cardiac dysfunction *in vivo* in postnatal life. Then we investigated whether PNE enhanced cardiac miR-181a expression, altered cardiac ATR gene methylation, H19 lncRNA expression and cardiac ischemia-sensitive signaling proteins (such as ATR, TGF-β/Smads, and autophagy-related genes) in adult offspring. Finally, we examined whether inhibition of miR-181a *via* miRNA antisense reversed nicotine-mediated cardiac ATR gene hypomethylation, leading to rescue of nicotine-mediated alterations of H19/ATR/TGF-β/Smads/autophagy signaling pathways, and consequently attenuated I/R-induced heart injury and dysfunction in the offspring.

## Methods

### Experimental animals

All of the procedures and protocols (IACUC#8170045) in present study were approved by the Institutional Animal Care and Use Committee of Loma Linda University and followed the guidelines of the NIH (National Institutes of Health, USA) Guide for the Care and Use of Laboratory Animals. Time-dated (day 2 of gestation) pregnant Sprague-Dawley rats were purchased from Charles River Laboratories (Portage, MI) and housed individually in plexiglas acrylic plastic cages located in air-conditioned rooms (room temperature 22 °C , relative humidity 60%; lights on from 8:00 a.m. to 8:00 p.m.). Pellet food and tap water were available ad libitum. On day 4 of gestation, the rats were randomly divided into two groups: 1) saline control and 2) nicotine-treated group. For the nicotine-treated group, rats were administered nicotine subcutaneously at 4 μg/kg/min via an osmotic minipump implanted from gestational day 4 until postnatal day 10. The rationale for selecting this dosage is that it produces maternal plasma nicotine levels closely resembling those occurring in moderate human smokers 21. The rationale for nicotine treatment from gestational day 4 until postnatal day 10 is that it is the important period starting right before the embryos implant in the rat uterus until the neonatal heart near full development, which allow us to see how nicotine exposure affects fetal and neonatal heart development. For the control group, rats received an equal volume of saline from the osmotic minipump as the vehicle control. The following experiments were conducted in their offspring at about three months of age.

### Echocardiography measurement

Cardiac function in the offspring from each group was measured by echocardiography (GE Healthcare, USA). Echocardiography assessment was conducted 4 times for each rat including Baseline (10 days before I/R), 1 day before I/R, 1 day after I/R, and 7 days after I/R. Briefly, offspring were anesthetized with inhalation of 3.5% isoflurane and placed on a pre-warmed (37 °C) work surface. The rats were shaved in the thorax area and placed in the left lateral decubitus position, then applied a layer of acoustic-coupling gel to the chest. M-mode recording of the left ventricle was obtained at the level of the mitral valve in the parasternal view using two-dimensional (2D) echocardiographic guidance in both short and long axis views. Cardiac function and heart dimensions were evaluated by 2D echocardiography on the anesthetized (2% isoflurane) rat. M-mode tracing was used to measure functional parameters such as LV end-diastolic dimension (LVEDD), LV end-systolic dimension (LVESD), LV end-diastolic volume (LVEDV) and LV end-systolic volume (LVESV) were calculated using the above primary measurements and accompanying software. The percentage of LV ejection fraction (EF) was calculated as: (LVEDV-LVESV)/LVEDV × 100% and the percentage of LV fractional shortening (FS) was calculated as: (LVEDD-LVESD)/LVEDD × 100%. Echocardiographic data were recorded and analyzed blindly to the treatment groups.

### Heart ischemic-reperfusion (I/R) model and measurement of myocardial infarct size

The offspring rats (~3 month-old) were subjected to myocardial ischemia-reperfusion *in vivo* as described previously 22. Briefly, rats were anesthetized with isoflurane (5% for induction, 3% for maintenance) (Vet ONE, USA) mixed with oxygen (2 L/min for induction, 1 L/min for maintenance) by inhalation and placed on the RoVent Jr. Small Animal Ventilator (Kent Scientific). The respiratory parameters of the mechanical ventilator were adjusted by the body weight of rats. The ischemia was induced by ligation of the left anterior descending artery (LAD) with a 6-0 PROLENE® suture (Ethicon, USA) for 45 min, after which the suture was removed to achieve reperfusion. After surgery, offspring were recuperated in a single cage for 20 min before they returned to their house cages.

For measurement of cardiac infarct size, the whole hearts were collected and cut into 4 slices on ice, after 24 h of I/R. Then, heart slices were incubated in 2% triphenyl tetrazolium chloride (TTC) (Sigma-Aldrich, USA) solution for 10 min at 37 °C away from light and immersed in formalin (Thermo Scientific, USA) over-night. Each slice was photographed and the areas of myocardial infraction size and LV were analyzed by Image J (National Institutes of Health, USA), which is a java-based image processing program. The size of myocardial infarct was expressed as the ratio of myocardial infarct size to whole LV area, as previously described 23.

### *In vivo* treatment of LNA-miR-181a

In order to determine the functional significance of the enhanced miR-181a expression patterns in nicotine-mediated heart I/R injury in adult offspring, miR-181a inhibitor was administered to both the saline control and nicotine-exposed male offspring ten days before I/R with locked nucleic acid antisense oligonucleotides specific for miR-181a (LNA-miR-181a) (10 mg/kg × 2 every 5 days, i.p.). LNA-anti-miRNAs are widely used for inhibition of miRNAs in different animal models 24-26. The rationale for selecting this dosage is based on a previous study 26, in which intraperitoneal injection of a similar dosage of LNA-miR-181a can significantly inhibit miR-181a levels and improves rodent liver function. The LNA-miR-181a working solutions (50 mg/ml in saline) were prepared according to manufacturer's instructions (Qiagen Inc.). In addition, in another set of experiments, scrambled LNA-miR-181a (10 mg/kg × 2 every 5 days, i.p.) and saline solution were administered to ~ 3 month-old male rats to see if the scrambled negative control of miR-181a affects miR-181a expression and cardiac function. After ten days of treatment, the rats from both the saline control and nicotine-exposed groups underwent the cardiac I/R procedure. Echocardiography was performed at the following time points: 1) before LNA-miR-181a treatment (baseline), 2) one day before I/R (ten days after LNA-miR-181a treatment), 3) one day after I/R, and 4) seven days after I/R, respectively. The left ventricle tissues at the indicated time points were isolated from each group for infarct analysis and other molecular biological studies.

### qRT-PCR quantification

Cardiac miR-181, H19, and AT_1_R/AT_2_R mRNA levels were measured by miScript II RT kit (Qiagen) and SYBR Green PCR kit (Qiagen) according to manufacturer's instructions. Primers for miR-181a, miR-181b, miR-181c, H19, and SNORD61 were obtained from miScript Primer Assay kit (Qiagen). The primers for AT_1a_R, AT_2_R and GAPDH were designed and obtained from IDT Inc. AT_1_aR: 5′-GGAGAGGATTCGTGGCTTGAG-3′ (sense) and 5′-CTTTCTGGGAGGGTTGTGTGAT-3′ (antisense); AT_2_R: 5′-CAATCTGGCTGTGGCTGACTT-3′ (sense) and 5′-TGCACATCACAGGTCCAAAGA-3′ (antisense); GAPDH: 5′-TGACTCTACCCACGGCAAGTTCAA-3′ (sense) and 5′-ACGACATACTCAGCACCAGCATCA-3′ (antisense). Briefly, template RNA isolated from a 1-2 mm slice from the apex of the left ventricle was mixed with reverse-transcription master mix in a final volume of 20 µl and incubated for 60 min at 37 °C, and the reaction was stopped at 95 °C. Converted cDNA were used for quantification in a final volume of 20 µl system containing specific primers and QuantiTect SYBR Green PCR master mix following manufacturer's instructions. PCR threshold cycle (CT) numbers were averaged for each sample. Comparative CT method (ΔΔCt) was used for the RT-qPCR data analysis. The relative miR-181a/b/c levels were calculated as the Ct values of miR-181a/b/c to the reference gene (SONORD61) and the relative mRNA levels of AT_1_aR/AT_2_R and H19 were calculated as the Ct values of respective primer to the reference gene (GAPDH).

### Bisulfite conversion of genomic DNA and quantitative methylation-specific PCR (MSP)

Genomic DNA was isolated from a 1-2 mm slice from the apex of the left ventricle with A260/280 >1.6 and bisulfite‐converted using a BisulFlash DNA modification kit (Epigentek, Farmingdale, NY, USA). PCR Bisulfite-treated DNA was used as a template for real-time fluorogenic methylation-specific PCR (MSP). Bioinformatics analyses of AT1R/AT2R promoter sequence were based on AT_1_aR: Gene ID: 24180, updated on 31-Dec-2019; and AT_2_R Gene ID: 24182, updated on 16-Dec-2019. AT_1_R oligo primers are listed as follow, C/EBP-δ(-133)-M-sense: 5'-TTGAATTGTTAGTGAGGATTC-3'; UM-sense: 5'-TTGAATTGTTAGTGAGGATT-3'; antisense: 5'-AAAACCAAAATCACTCTAATACTC-3'; TAF(-247) M-sense: 5'-GAGTATTAGAGTGATTTTGGTTTTC-3'; UM-sense: 5'-GAGTATTAGAGTGATTTTGGTTTTT-3'; antisense: 5'-AAAAAAAAAAAAAAAAAAAAAAAAAAAAAA-3'; C/EBP-α(-505)-M-sense: 5'-TTTTTATTTTTAAATAAATGTAGTTC-3'; UM-sense: 5'-TTTTTATTTTTAAATAAATGTAGTTT-3'; antisense: 5'-TAACTCAAAATATAATACCT-3'; ZF-5(-838)-sense: 5'-GTTTATGTGGTTTTATGTTTATT-3'; M-antisense: 5'-CTCCCTCTCTAATTTCTAACG-3', UM-antisense: 5'-CTCCCTCTCAATTTCTAACA-3'; Sp-1(-886)-sense: 5'-ATATTGGGTGATTGGTAGTAG-3', M-antisense: 5'-ACTTTACACTCCTCCCCCG-3', UM-antisense: 5'-ACTTTACACTCCTCCCCCA-3'. AT2T primers list as followed, Pax-6 (-395)-M-sense: 5'-TTAGAAAGTATATAGATGGAAAAATCGT-3', M-antisense: 5'-ATAAAAACTAATCAAAATAAACGAC-3', UM-sense: 5'-TTAGAAAGTATATAGATGGAAAAATTGT-3', UM-antisense: 5'-ATAAAAACTAATCAAAATAAACAAC-3'; Msx-1(-71)-sense: 5′-TTTTTTGGAAAGTTGGTAAGTGTTTA-3′; M-antisense: 5′-CTCTAATTTCCTTCTTATATATTCG-3′, UM-antisense: 5′-CTCTAATTTCCTTCTTATATATTCA-3′; and ZF5(+71)-sense: 5′-GAAGGTTTTTTAGTGGATAG-3′; M-antisense: 5′-AAAAAAAACTTTCAATTCTATACTCG-3′; UM-antisense: 5′-AAAAAAAACTTTCAATTCTATACTCA-3′; qMSP was performed using SYBR green qPCR mix (bimake). Each amplification reaction was performed in duplicate or triplicate in a volume of 20 µl containing 2.5 ng bisulfite‐modified DNA. The methylation index (MI), representing the ratio of densely methylated DNA in the sample at the target sequence calculated as the ratio of methylated DNA Ct values to the sum of Ct values of methylated DNA and unmethylated DNA.

### Western blot analysis

Total protein was isolated from samples of the apex of left ventricle of the offspring and were homogenized in lysis buffer containing 150 mM NaCl (Bio-Rad, USA), 50 mM Tris·HCl (Bio-Rad, USA), 10 mM EDTA (Bio-Rad, USA), 0.1% Tween (Fisher, USA), 0.1% β-mercaptoethanol (Bio-Rad, USA), 0.1 mM phenylmethylsulfonyl fluoride (Bio-Rad, USA), 5 ug/mL leupeptin (Bio-Rad, USA), and 5 ug/mL aprotinin (Bio-Rad, USA), pH 7.4. Then, the homogenates were centrifuged at 4 °C for 15 min at 12,000 g and the supernatants were collected. Samples with equal amounts of total protein were loaded in the 8% or 10% polyacrylamide gel with 0.1% SDS (Bio-Rad, USA) and were separated by electrophoresis at 100 V for 80 min. Proteins were then transferred onto nitrocellulose membranes (Bio-rad, USA) and blocked for 3 h at room temperature. The membranes were incubated at 4 °C with primary antibodies against AT_1_R, AT_2_R, TGF-β, smad2/3, smad4, smad5, smad6, samd7, Beclin1, Atg5, and LC3B, respectively. All of these primary antibodies were obtained from Cell Signaling Technology, USA. Primary antibody against GAPDH (MilliporeSigma, USA) served as an internal control. After washing and incubating with secondary antibodies, protein bands were visualized with enhanced chemiluminescence reagents and captured by photographic films (MidSci, USA). The films were analyzed with a document imaging scanner (HP, USA) with gray mode. Band intensities were normalized to GAPDH.

### Statistical analysis

All data are expressed as the mean ± SEM obtained from the number (n) of experimental animals given. Difference between the groups was compared by Student's *t*-test or analysis of variance (ANOVA) using the Graph-Pad Prism software (GraphPad Software Version 6, San Diego, CA, USA) or SPSS software 19 (SAS, NC) wherever appropriate. For all comparisons, *P*-values less than 0.05 indicated statistical significance.

## Results

### PNE impaired heart function and enhanced I/R-induced heart infarction in gender dependent manner

**Figure 1:**
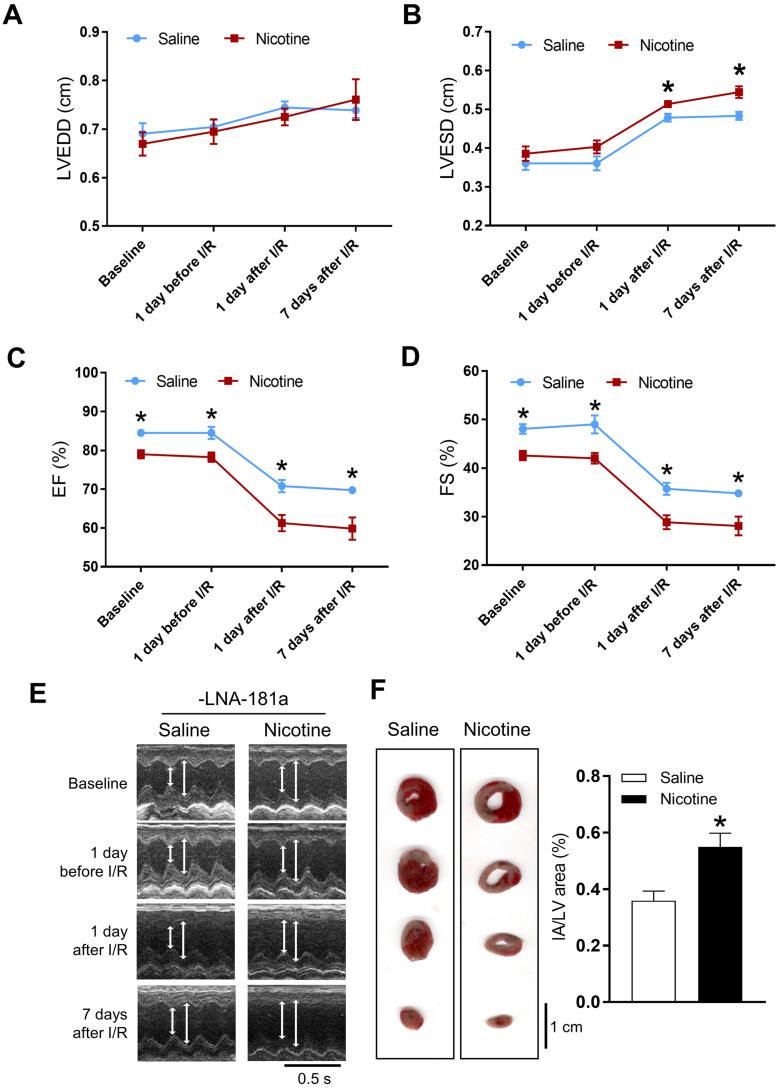
** Effects of PNE on heart function and ischemia-reperfusion induced heart infarct size.** Rats were administered with either saline or nicotine from gestation day 4 until postnatal day 10. The offspring at 3-month-old age from each group were subjected to 45 min of heart ischemia followed by reperfusion. Echocardiographic analysis was obtained from different time periods including baseline, 1 day before I/R, 1 day after I/R, and 7 days after I/R. (**A**) left ventricular end-diastolic dimension (LVEDD), (**B**) left ventricular end-systolic dimension (LVESD), (**C**) percentage of ejection fraction (EF%), (**D**) percentage of fractional shortening (FS%), (**E**) representative echocardiograph evaluation of cardiac function from each group (n=3~10 animals/group), *P < 0.05 versus saline control group, as determined by Student's t-test. For the infarction size, the hearts of rats were isolated 24 h after I/R and their infarct sizes in each rat group were determined with 2% TTC staining (**F**), the graph showing the percentage of left ventricle infarct size (infarct area/ left ventricle area x100%) in each offspring group (n=4~5 animals/group). *P < 0.05 versus saline control group, as determined by Student's *t*-test.

Cardiac functions at the baseline and post I/R were measured by echocardiography. In the female offspring, there were no significant differences of the baseline heart function between the saline and nicotine-exposed groups (Table S1 and Figure S2). In addition, there were also no differences of the heart function on the first day after I/R, but increases in LVEDV and SV were observed on the 7th day after I/R in the female offspring (Table S1). Furthermore, PNE had no effect on the I/R-induced myocardial infarct size in the female offspring (Figure S1). In contrast, the cardiac functions in the male offspring were significantly affected by PNE at both the baseline and post I/R. As shown in Table S2 and Figure 1, nicotine exposure differentially decreased the values of IVSdd, IVSsd, LVPWs, EF, FS, and SV as compared to the controls at the baseline. Furthermore, nicotine exposure also altered the values of LVESD and LVESV in response to the I/R procedure (Table S2). In addition, there was a substantially higher cardiac infarct size in the nicotine exposed group than in the control group in the male offspring 24 h after the I/R procedure (Figure 1F).

### PNE up-regulated cardiac miR-181a expression and LNA-miR-181a reversed PNE-mediated heart function and I/R-induced cardiac infarction in male offspring

**Figure 2:**
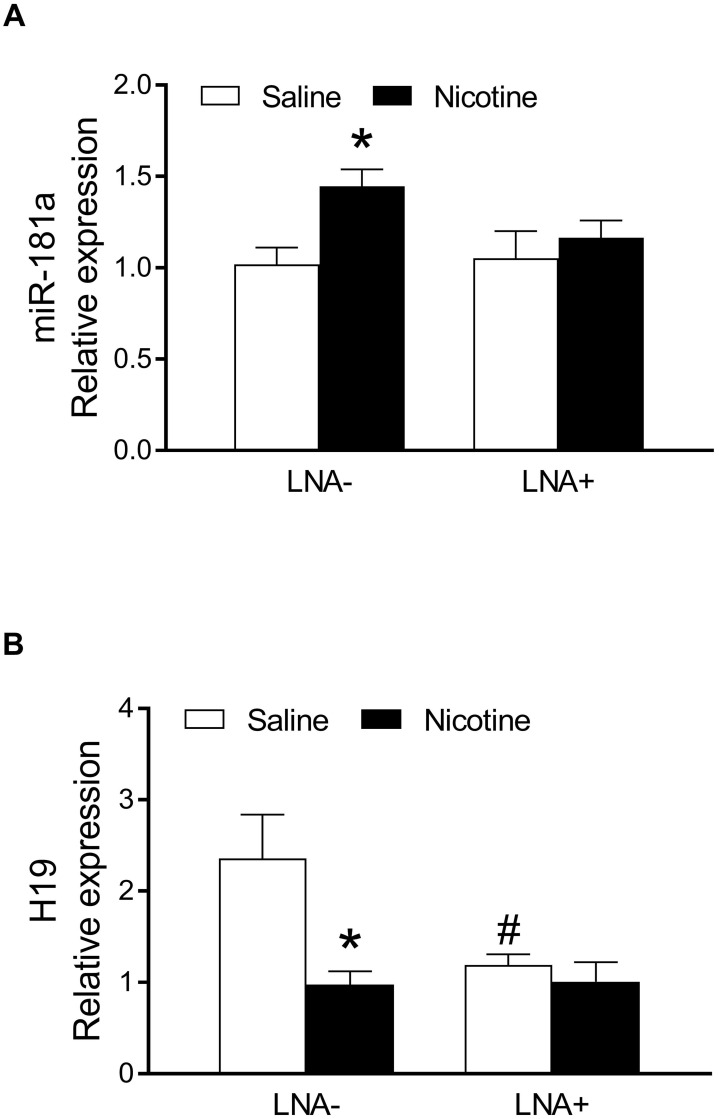
** Effects of PNE on miR-181a and H19 levels in offspring rats.** The total RNA samples were isolated from left ventricle tissues of the male offspring from each group and the cardiac miR-181a and H19 levels were determined by qRT-PCR analysis as described in the Material and Methods. (**A**) miR-181a levels of each group (n=6 animals/group), (**B**) H19 levels of each group (n=7 animals/group). Data are means ± SEM. The expression levels of miR-181a are normalized by SNOTD61. *P < 0.05 versus saline control group, as determined by Student's *t*-test and the expression levels of H19 are normalized by GAPDH. *P < 0.05 versus saline control group, #*P* <0.05 vs. without LNA-miR-181a treatment, as determined by two-way ANOVA test.

Since nicotine exposure has a profound effect on heart function and I/R-induced heart injury in male but not female offspring, we focused mainly on the underlying molecular mechanisms in male offspring. As shown in Figure 2, by measurement of miR-181 levels in the heart of the male offspring *via* qRT-PCR analysis, a significant up regulation of miR181a levels was observed in the nicotine-exposed group as compared to the saline control group (Figure 2A). However, there is no significant difference of miR-181a levels between the saline control and nicotine-exposed groups after treatment with LNA-miR-181a (Figure 2A). In addition, LNA-miR-181a scramble treatment had no significant effect on miR-181a levels (Figure S4A). Furthermore, we also measured the miRNA levels of other miR-181 family members. As shown in the Figure S4, nicotine exposure had no effect on miR-181b (Figure S4B) but significantly enhanced miR-181c (Figure S4C) levels as compared to the controls, and the enhanced miR-181c levels were not affected by LNA-miR-181a treatment (Figure S4C).

**Figure 3:**
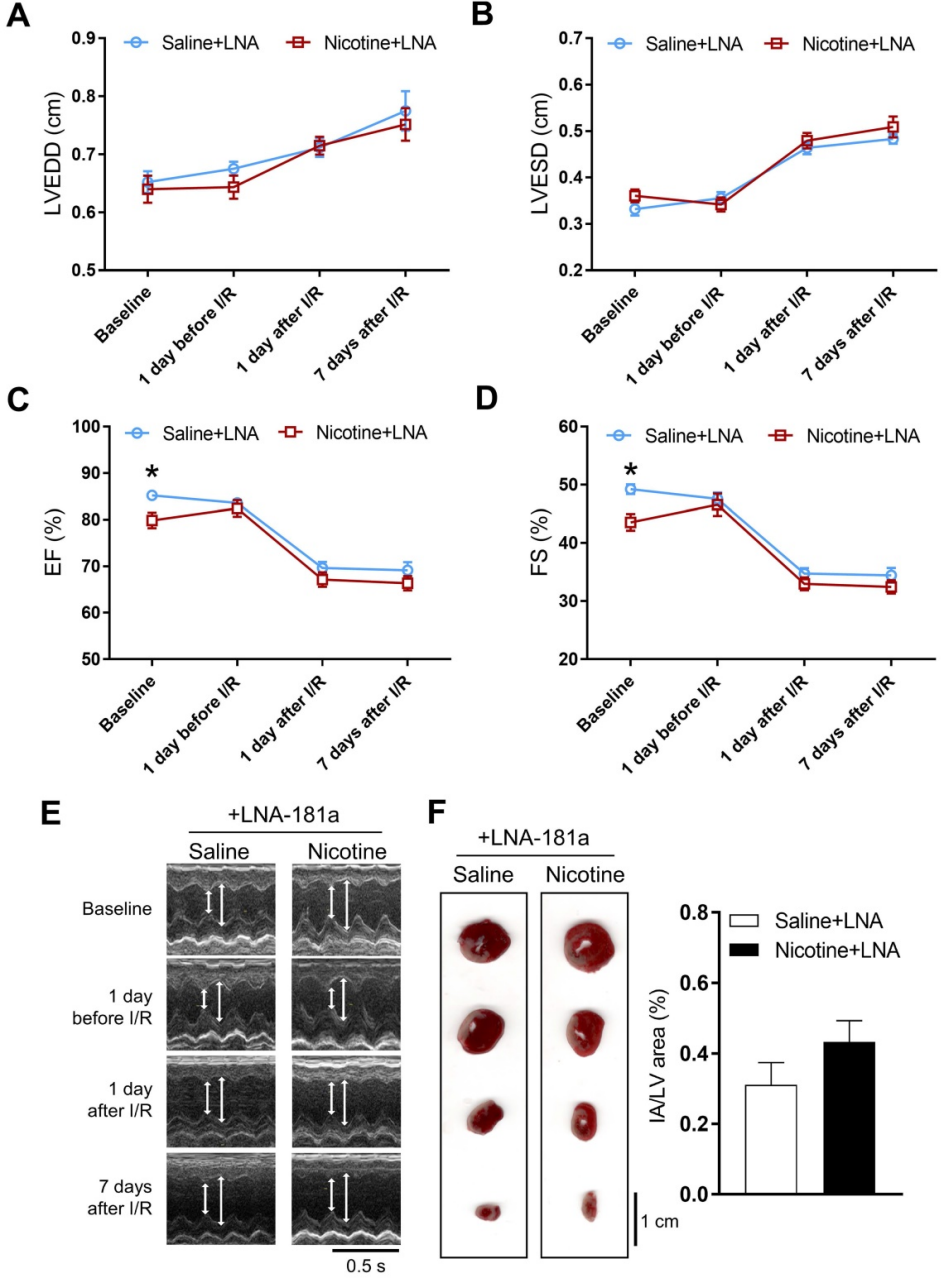
** Effects of PNE on heart function and ischemia-reperfusion induced heart infarct size after treatment of LNA-miR-181a.** Rats were administered with either saline or nicotine from gestation day 4 until postnatal day 10. After treatment with LNA-miR-181a, the offspring at 3-month-old age from each group were subjected to 45 min of heart ischemia followed by reperfusion. Echocardiographic analysis was obtained from different time periods including baseline, 1 day before I/R, 1 day after I/R, and 7 days after I/R. (**A**) left ventricular end-diastolic dimension (LVEDD), (**B**) left ventricular end-systolic dimension (LVESD), (**C**) percentage of ejection fraction (EF%), (**D**) percentage of fractional shortening (FS%), (**E**) representative echocardiograph evaluation of cardiac function from each group (n=4~10 animals/group), *P < 0.05 versus saline control group, as determined by Student's t-test. For the infarction size, the hearts of rats were isolated 24 h after I/R and their infarct sizes in each rat group were determined with 2% TTC staining (**F**), the graph showing percent of left ventricle infarct size (infarct area/ left ventricle area x100%) in each offspring group (n=4 animals/group). *P < 0.05 versus saline control group, as determined by Student's *t*-test.

In order to see whether the nicotine-enhanced miR-181a plays a key role in nicotine-mediated cardiac dysfunction in the male offspring, male offspring from both saline control and nicotine-exposed groups were treated with LNA-miR-181a for ten days before I/R procedure. As shown in Table S3 and Figure 3, the differences of EF, FS, SV, and LVPWs between the saline control and nicotine-exposed groups were abolished by LNA-miR-181a treatment before I/R. In addition, the nicotine-mediated changes of IVSdd were not altered by LNA-miR-181a treatment both before and after I/R (Table S3). However, LNA-miR-181a treatment did not affect nicotine-mediated changes of IVSsd before I/R but attenuated the nicotine's effect after I/R (Table S3). Furthermore, treatment with LNA-miR-181a eliminated the difference of infract size between the saline control and nicotine-treated groups following I/R (Figure 3F). In addition, we did additional control experiments with scrambled LNA-miR-181a treatment. As shown in Table S4 and Figure S3, pretreatment with the scrambled LNA-miR-181a at the same concentration of LNA-miR-181a antisense had no significant effect on cardiac function as compared to the saline controls in each period of time. This data suggests that scrambled LNA-181a has no significant physiological effect on cardiac function.

### PNE down-regulated on lncRNA H19 expression in the left ventricle tissues

In order to determine whether PNE alters lncRNA H19 levels in cardiac tissues of male offspring, qRT-PCR analysis was used to measure the cardiac H19 levels in each group of animals. As shown in Figure 2B, the lncRNA H19 levels in the left ventricle tissues were substantially down regulated in the nicotine-exposed group as compared to the saline-control group in the absence of LNA-miR181a. However, the levels of H19 in LV tissues were not significantly different between the saline control and nicotine-exposed offspring with the LNA-miR-181a treatment. Interestingly, LNA-miR181a significantly decreased H19 levels in the saline-control group but not in the nicotine-exposed group.

### PNE down-regulated specific CpG methylation pattern of AT_1_R/AT_2_R promoter in the left ventricle tissues

**Figure 4:**
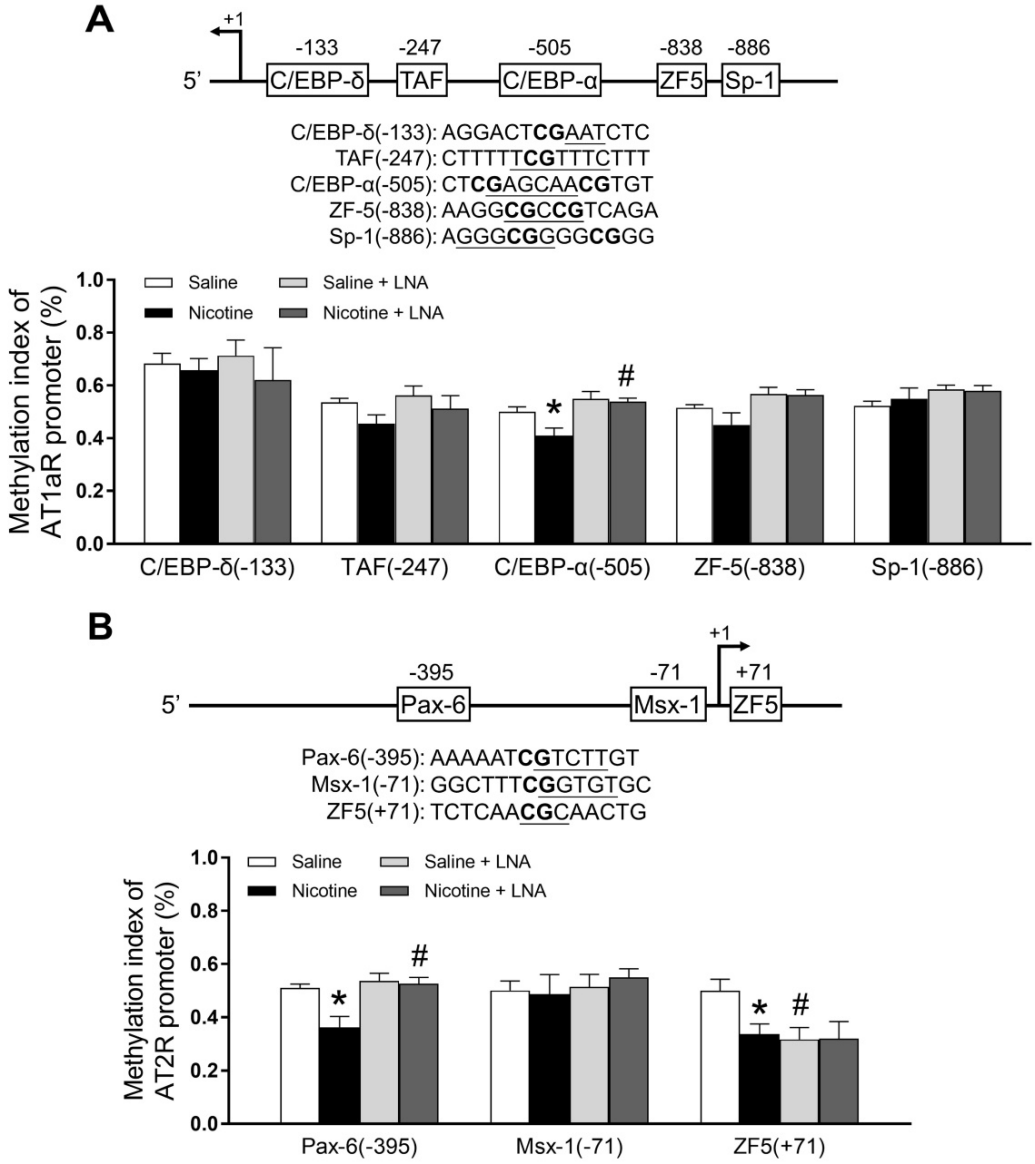
** Effects of PNE on DNA methylation of AT_1_R and AT_2_R promoter in male offspring.** Left ventricle tissues were freshly isolated from male offspring that had been perinatally exposed to saline and nicotine. DNA sample isolated and methylation levels were determined by methylation specific PCR analysis. (**A**) Methylation levels in AT_1_R promoter region. (**B**) Methylation levels in AT_2_R promoter region. Data are means ± SEM (n=4~5 animals/group). *P < 0.05 vs. control, #*P* <0.05 vs. without LNA-miR-181a treatment, as determined by two-way ANOVA test.

Bioinformatics analysis of AT_1_R/AT_2_R promoter sequence identified several putative transcription factor binding sites that contained CpG dinucleotides in or near their core binding sequences, including C/EBP-δ on -133, TAF on -247, C/EBP-α on -505, ZF5 on -838, Sp-1 on -886 in AT_1_/R promoter (Figure 4A); Pax-6 on -395, Msx-1 on -71 and ZF5 on +71 in AT_2_/R promoter (Figure 4B). CpG methylation patterns at these binding sites were determined by quantitative methylation-specific PCR. As shown in Figure 4A, PNE selectively decreased CpG methylation level of the AT_1_R promoter at the C/EBP-α-505 binding site, whereas LNA-miR-181a treatment increased its CpG methylation levels in the nicotine-exposed group and eliminated the difference between the saline control and nicotine- exposed groups. As shown in Figure 4B, PNE selectively decreased CpG methylation levels of the AT_2_R promoter at both Pax-6 (-395) and ZF-5 (+71) binding sites. LNA-miR-181a treatment enhanced CpG methylation levels at the PAX-6 binding site in the nicotine-exposed group and eliminated its difference between the saline control and nicotine-exposed groups. On the other hand, LNA-miR-181a treatment decreased the CpG methylation level at ZF-5 (+71) binding site in the saline control group and eliminated its difference between the saline control and nicotine-exposed groups.

### PNE enhanced cardiac angiotensin II receptor (ATR) protein and mRNA expression

**Figure 5:**
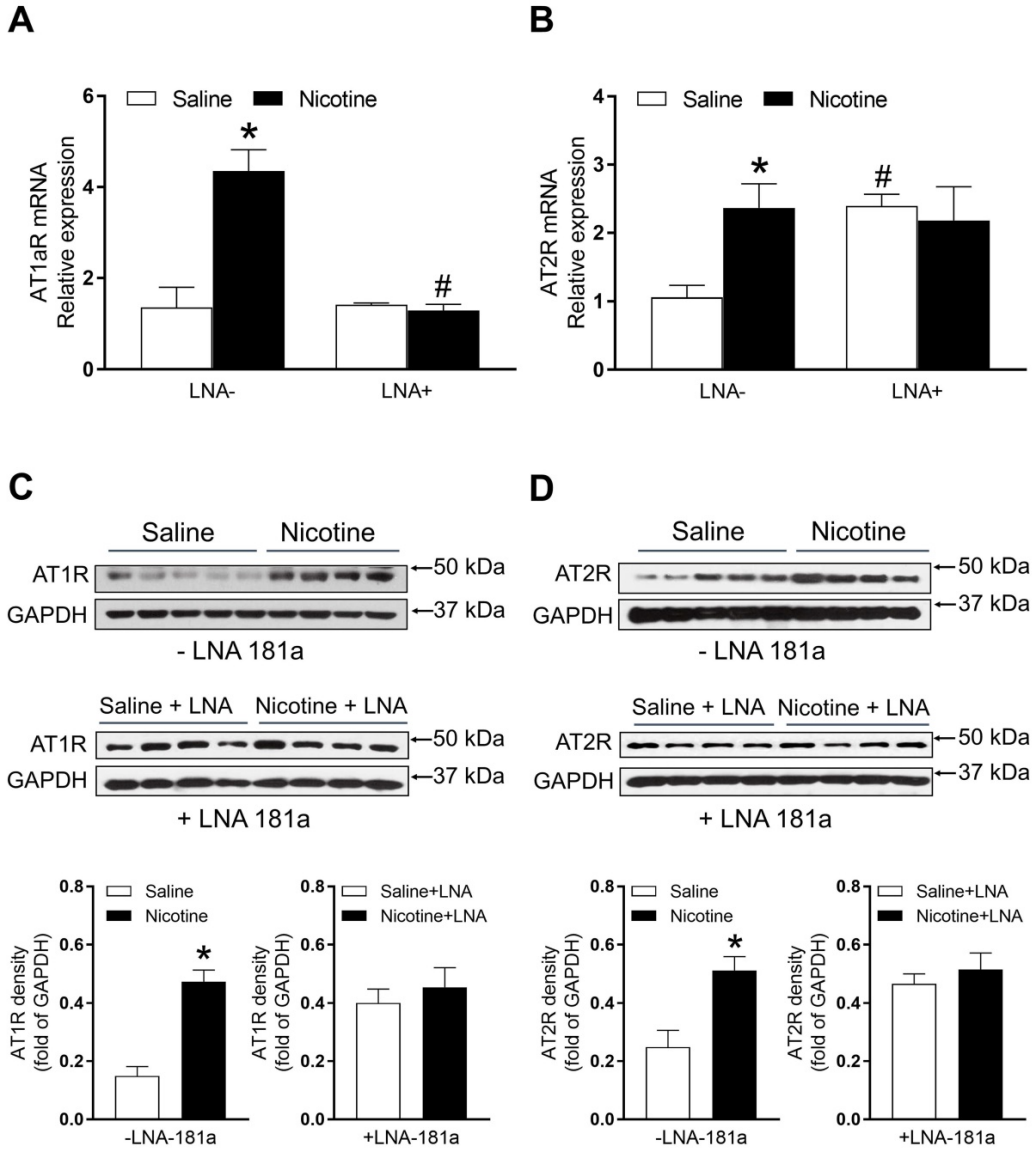
** Effects of PNE on AT_1_R and AT_2_R mRNA and protein expression in male offspring.** Left ventricle tissues were freshly isolated from male offspring that had been perinatally exposed to saline and nicotine. The cardiac mRNA levels of AT_1_R (**A**) and AT_2_R (**B**) were measured by qRT-PCR analysis, the mRNA expression levels of AT_1_R and AT_2_R are both normalized by GAPDH. *P < 0.05 vs. saline control group, #*P* <0.05 vs. without LNA, as determined by two-way ANOVA test. The cardiac protein levels of AT_1_R (**C**) and AT_2_R (**D**) were determined by Western blot analysis. Their protein densities are normalized as fold of GADPH density. Data are means ± SEM (n=4~5 animals/groups). *P < 0.05 vs. control, as determined by Student's *t*-test.

As shown in Figure 5A, PNE significantly enhanced mRNA levels of AT_1_aR as compared to the saline control group. LNA-miR-181a treatment significantly attenuated the AT_1_aR mRNA levels in the nicotine-exposed group and eliminated its differences between the two groups (Figure 5A). Similarly, PNE also significantly enhanced AT_2_R mRNA levels compared to the saline control group (Figure 5B). However, LNA-miR-181a treatment significantly enhanced AT_2_R mRNA levels in the saline group and eliminated its differences between the two groups (Figure 5B).

The protein levels of AT_1_R and AT_2_R in the left ventricle tissues were determined by Western blot analysis. As shown in Figure 5C and D, PNE significantly increased the protein levels of both AT_1_R and AT_2_R in LV tissues as compared with the saline control group in the absence of LNA-miR-181a treatment. However, in the presence of LNA-miR-181a, the protein levels of both AT_1_R and AT_2_R in LV tissues were not significantly different between the saline control and nicotine-exposed groups.

### PNE differentially increased protein abundances of TGF-β and SMAD in the left ventricle tissues

**Figure 6:**
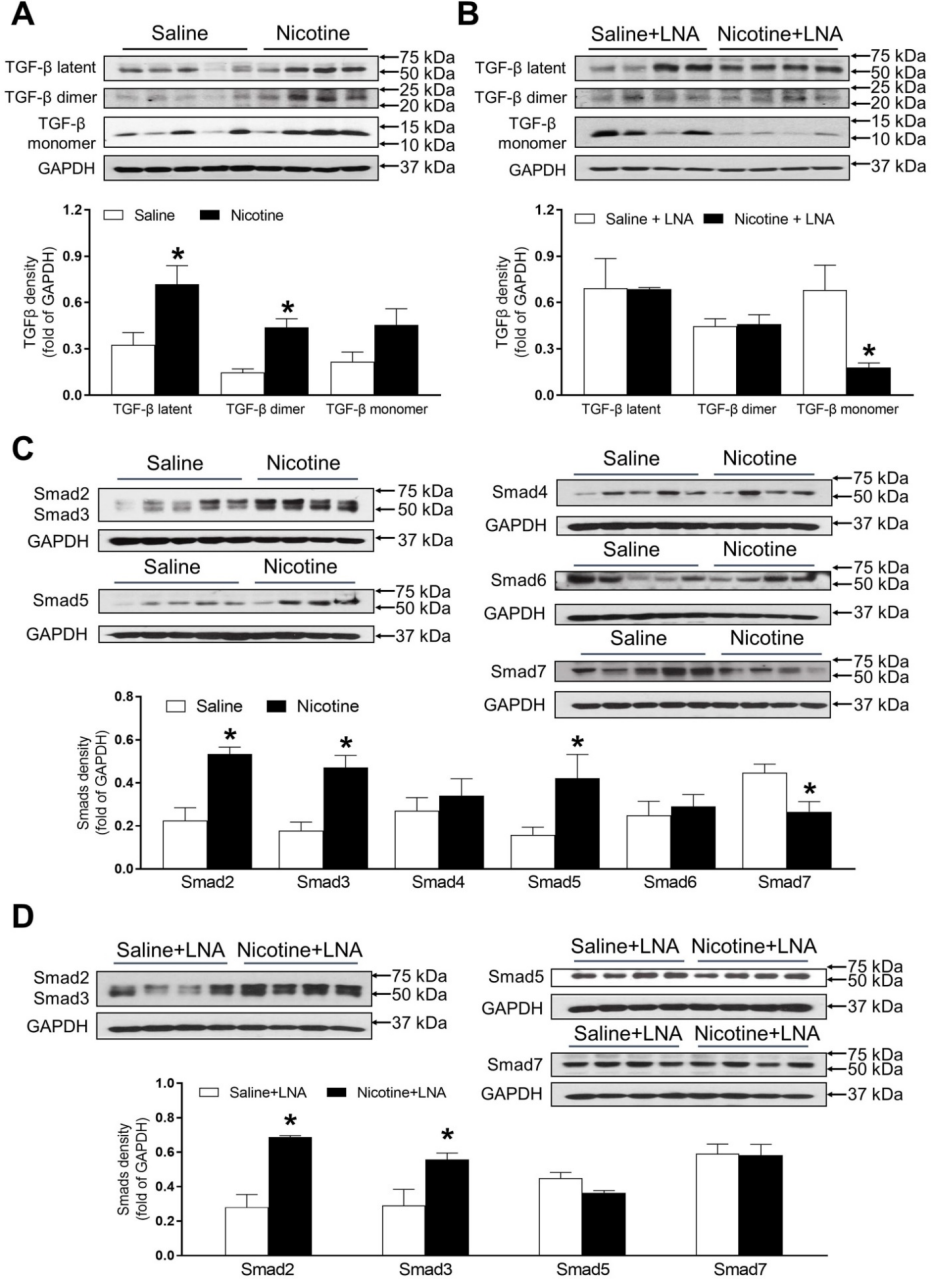
** Effects of PNE on TGF-β and Smad protein expression in male offspring.** Left ventricle tissues were freshly isolated from male offspring that had been perinatally exposed to saline and nicotine. The protein levels of TGF-β isoforms and Smads family were determined by Western blot analysis and were normalized as fold of GAPDH density. (**A**) TGF-β protein density in the absence of LNA-miR-181a, (**B**) TGF-β protein density in the presence of LNA-miR-181a, (**C**) Smads protein density in the absence of LNA-miR-181a, (**D**) Smads protein density in the presence of LNA-miR-181a. Data are means ± SEM (n=4~5 animals/groups). *P < 0.05 vs. control, as determined by Student's *t*-test.

As shown in Figure 6A, PNE remarkably up regulated the offspring cardiac protein abundances of TGF-β latent complex and mature TGF-β dimer as compared with the saline control group in the absence of LNA-miR-181a treatment. Whereas, there were no significant differences of the protein levels of TGF-β latent complex and mature TGF-β dimer between the nicotine-treated and saline control offspring after the treatment of LNA-miR-181a (Figure 6B). In addition, PNE did not significantly affect the levels of TGF-β monomer expression as compared with the saline control group in the absence of LNA-miR-181a treatment (Figure 6A). Interestingly, with the LNA-miR-181a treatment, TGF-β monomer expression dramatically declined in the nicotine-treated group compared to the saline control group (Figure 6B).

As shown in Figure 6C, PNE differentially increased the cardiac protein levels of Smad2, Smad3 and Smad5 but significantly decreased Smad7 protein levels as compared to the saline control group in the absence of LNA-miR-181a treatment. In addition, there were no significant differences in Smad4 or Smad6 levels between the two groups (Figure 6C). On the other hand, treatment with LNA-miR-181a had no effect on nicotine-mediated changes of Smad2 and Smad3 but eliminated the differences of Smad5 and Smad7 protein expression between the saline control and nicotine-exposed groups (Figure 6D).

### PNE increased the protein abundances of autophagy-associated biomarkers in the left ventricle tissues

**Figure 7:**
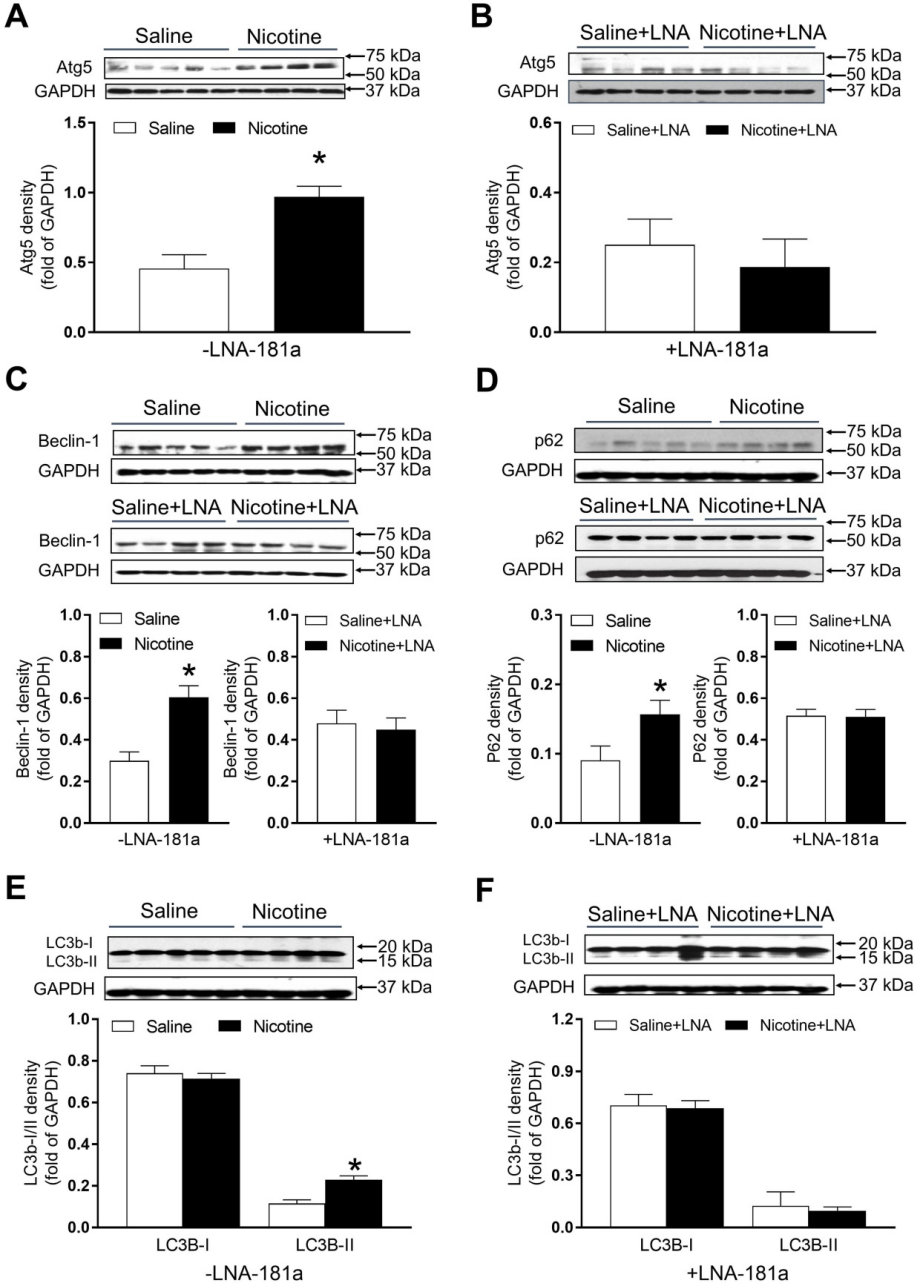
** Effects of PNE on autophagy protein expression in male offspring.** Left ventricle tissues were freshly isolated from male offspring that had been perinatally exposed to saline and nicotine. The protein levels of autophagy-related genes were determined by Western blot analysis and were normalized as fold of GAPDH density. (**A**) Atg-5 protein density in the absence of LNA-miR-181a, (**B**) Atg-5 protein density in the presence of LNA-miR-181a, (**C**) Beclin-1 protein density of each group, (**D**) p62 protein density of each group, (**E**) LC3B-I and LC3B-II protein density in the absence of LNA-miR-181a, (**F**) LC3B-I and LC3B-II protein density in the presence of LNA-miR-181a. Data are means ± SEM (n=4~5 animals/groups). *P < 0.05 vs. control, as determined by Student's *t*-test.

As shown in Figure 7, there were significant increases in the autophagy-related protein Atg-5 (Figure 7A), Beclin1 (Figure 7C) and p62 (Figure 7D) expression levels in the cardiac tissues of PNE offspring as compared to the saline controls in the absence of LNA-miR-181a. However, in the presence of LNA-miR-181a, protein levels of Atg-5 (Figure 7B), Beclin1 (Figure 7C) and p62 (Figure 7D) in the LV tissues were not significantly different between the two groups. Similarly, the protein expressions of LC3B II but not LC3 I in the LV tissues were higher in the nicotine-treated group than those in the saline control group (Figure 7E), which were rescued by the treatment of LNA-miR-181a (Figure 7F).

## Discussion and Conclusion

Epidemiological and animal studies have revealed that maternal tobacco smoking is positively and robustly associated with an increased risk of cardiovascular disease in offspring 27. The present study further demonstrates that PNE-mediated reprogramming of miR-181a expression patterns plays a crucial role in the cardiac dysfunction in adult male offspring. The major findings in the present study are: 1) PNE caused cardiac dysfunction and increased cardiac I/R infarct size in postnatal life, whereby nicotine exposure had a more predominant effect on male than on female offspring heart function; 2) PNE induced an up-regulation of miR-181a but down-regulation of H19 expression, which were associated with increased ATR protein expressions *via* specific CpG hypomethylation at the ATR gene promoter region; 3) PNE also differentially enhanced levels of TGF-β/Smads signaling molecules and autophagic flux in the male offspring cardiac tissues; 4) inhibition of miR-181 with a LNA-miR-181a normalized the aberrant miR-181a/H19 expression and CpG hypomethylation at the ATR promoter, and furthermore eliminated the differences in abundances of ATR protein, TGF-β/Smads signaling molecules, and autophagic flux between the saline control and nicotine-exposed groups; 5) finally, treatment with LNA-miR-181a attenuated nicotine-mediated I/R infarction and nicotine-mediated cardiac dysfunction. These findings suggest a potentially novel therapeutic strategy of miR-181a silencing for the treatment of cigarette smoking/nicotine-mediated heart ischemic disease.

Epidemiologic and animal studies have shown that maternal cigarette smoking/nicotine use during pregnancy is one of the most common causes of aberrant development of fetal heart and cardiovascular dysfunction in offspring 27, 28. Our previous studies in a pregnant rat model have demonstrated that PNE has no effect on pre-ischemic baseline values of cardiac function but enhances heart susceptibility to I/R injury and heart dysfunction in an *ex vivo* Langendorff preparation in adult male offspring 11,29,30. Similar to previous studies, our present study also demonstrated that PNE impaired cardiac function determined by *in vivo* echocardiography analysis in 3-month-old offspring. In the present study, we further found that PNE not only attenuated cardiac function (EF, FS and SV) after *in vivo* I/R but also impaired cardiac function before the I/R procedure in male offspring determined by echocardiography. The present data suggests that PNE not only affects heart function at normal physiologic conditions, but also increases the impairment of heart function when it encounters an ischemic stress challenge in male offspring. In addition, the present data that PNE enhanced I/R-induced LV infarction and cardiac dysfunction in male but not female offspring, suggests a gender difference in fetal programming of cardiovascular dysfunction in postnatal life. This is consistent with previous studies showing a gender-dependent alterations of vascular reactivity, blood pressure response, and brain ischemic injury in male but not female offspring in response to maternal nicotine exposure 31,32. Although the mechanisms underlying PNE-mediated gender difference of cardiac dysfunction in offspring are not fully understood, one of the potential mechanisms could be due to an intrinsic difference in the sex chromosome, or a sex dichotomy in the genes expressed in male and female placentas, or sex differences of fetuses in response to nicotine exposure. Of importance, previous studies suggest that sex hormones also play a significant role in the programming of cardiovascular disease in adulthood offspring 33. This opens a door for us in the future to investigate the molecular mechanisms that contribute to the sex difference of development of cardiac dysfunction in maternal nicotine-exposed offspring.

There is increasing evidence to support the important role of miRNAs in the development of cardiovascular disease 34. Recent studies have revealed that miR-181a expression is significantly increased during the progression of myocardial infarction in both animal models and patients 6-9. Consistent with these studies, present studies also showed that PNE-mediated cardiac ischemic injury and dysfunction is associated with an over-expression of miR-181a in LV tissues in male offspring. Most interesting, our recent studies have also demonstrated that miR-181a expression levels in offspring coronary vasculatures are enhanced in response to PNE 10. These findings suggest that the enhanced miR-181a could serve as a potential biomarker for offspring PNE-induced myocardial ischemic injury. However, currently we do not know whether PNE also alters miR-181a expression in other tissues/organs in the offspring. We need to further investigate this in our future studies. Furthermore, our results showed that treatment with LNA-miR-181a reversed nicotine-mediated miR-181a over-expression and attenuated nicotine-mediated cardiac dysfunction and I/R-induced infarct size in offspring. This suggests that reprogramming of miR-181a expression pattern in the cardiac tissue is one of the key mechanisms and contributors underlying perinatal nicotine-induced cardiac dysfunction and ischemic infarction. Similar to miR-181a expression, our current data also shows a significant increase in miR-181c expression in response to nicotine exposure. This finding opens the door for our future studies to see whether the over-expression of miR-181c plays a role in PNE-mediated cardiac dysfunctions.

Although our present data shows that PNE significantly enhanced miR-181a expression and treatment with LNA-miR-181a attenuated nicotine-mediated cardiac dysfunction and I/R-induced infarct size, the miR-181a regulatory signaling pathway may be only one of the contributors for PNE-mediated cardiac dysfunction. LncRNA H19 could be another important contributor. Recent studies have shown that lncRNA regulates tissue/cell homeostasis and plays a key role in pathological processes in heart development 12. Hypoxia stimulation down-regulated lncRNA H19 levels and was associated with attenuated proliferation and migration of cardiac progenitor cells 35. Additionally, in our present study, the finding that lncRNA H19 levels in the left ventricle tissues were substantially down-regulated in the nicotine-exposed group suggests that the decreased H19 may play a role in perinatal nicotine-mediated cardiac dysfunction. Indeed, previous studies have shown that H19 serves as a negative regulator of cardiomyocyte hypertrophy and cardiac dysfunction 36. The present finding that LNA-miR181a treatment inhibited H19 expression in the saline control group but not in the PNE group suggests that H19 expression can be regulated by miR-181a at normal physiologic condition, but that PNE-mediated H19 repression is not directly regulated through miR-181a signaling. Therefore, H19 repression could be another miR-181a-independent regulatory factor contributing to the PNE-induced cardiac dysfunction.

Previous studies have reported that cigarette smoking/nicotine exposure can alter renin-angiotensin system (RAS) in human and animal models 37, 38. Ang II mediates its effects *via* two receptors, AT_1_R and AT_2_R 16. There is clear evidence that the pathological process of cardiac disease is associated with alteration of ATR expression 16, 39. In our present study, PNE significantly increased both AT_1_R and AT_2_R protein and mRNA levels in the left ventricle as compared with the saline control group, which suggests that nicotine-mediated cardiac dysfunction may be associated with aberrant over-expression of the ATR. Our current findings demonstrate that treatment with LNA-miR-181a eliminated the differences in AT_1_R and AT_2_R gene expression between the saline control and nicotine-treated rats. These findings suggest that miR-181a is one of the key epigenetic up-stream regulators of ATR-mediated signaling in response to nicotine exposure. Our present findings that LNA-miR-181a treatment had no effect on AT_1a_R mRNA levels in the saline group but significantly decreased mRNA levels in the nicotine-exposed group, suggest that nicotine-mediated over-expression of AT_1a_R mRNA is mediated by miR-181a signaling. However, our present findings that LNA-miR-181a treatment significantly increased AT_2_R mRNA levels in the saline group but had no effect in the nicotine-exposed group, suggest that AT_2_R mRNA expression is negatively regulated by miR-181a signaling under physiological conditions, and that this negative regulation is blunted in response to perinatal nicotine exposure. Previous studies have suggested that nicotine could alter the homeostasis of the RAS by upregulating the detrimental angiotensin-converting enzyme (ACE)/Ang II/AT receptor axis, contributing to the development of cardiovascular and pulmonary diseases 40. In addition, nicotine could exacerbate cardiovascular remodeling-induced by Ang II treatment 41. Furthermore, it also has been demonstrated that angiotensin receptors contribute to the effects of nicotine on dopamine and norepinephrine release in brain regions involved in nicotine reward and hypertension 42. Taken together with our current findings, these reports support the notion that PNE could exacerbate cardiac ATR expression, leading to cardiac remodeling and development of an ischemia-sensitive heart in the offspring.

DNA methylation at gene promoter region is one of the important epigenetic mechanisms in the regulation of gene expression. To understand the epigenetic molecular mechanisms underlying perinatal nicotine-induced up-regulation of ATR gene expression in adult offspring, we further examined the specific CpG methylation patterns at the ATR gene promoter region. In our present study, we demonstrated that PNE selectively decreased levels of CpG methylation of the AT_1_R promoter at the C/EBP-α (-505) binding site, and selectively attenuated levels of CpG methylation of the AT_2_R promoter at PAX-6 (-395) and ZF5 (+71) binding sites. These findings suggest that nicotine-mediated AT_1_R/AT_2_R over-expressions are epigenetically regulated by CpG hypomethylation mechanisms in the offspring heart. Similarly, our previous study has demonstrated that PNE differentially alters specific CpG methylation levels in AT_1_R/AT_2_R promoter regions at various transcription factor (TF) binding sites 43. Alteration of CpG methylation at the binding sites of TFs could impair their binding affinity for binding the gene promoter, which could lead to a change gene promoter activity and protein expression. These studies further support that DNA methylation is a key epigenetic mechanism in regulation of gene expression in response to fetal stress. The present finding that LNA-miR-181a treatment rescued PNE-mediated CpG methylation levels of the AT_1_R promoter at the C/EBP-α (-505) binding site suggests that PNE-induced hypomethylation of the AT_1_R gene is directly regulated through the miR-181a signaling pathway. Of interest, our present data showed that LNA-miR-181a treatment enhanced the CpG methylation levels of the AT_2_R promoter at PAX-6 in the nicotine-exposed group but decreased CpG methylation levels at ZF-5 binding sites only in the saline group. This suggests that miR-181a could differentially regulate CpG methylation levels of the AT_2_R gene, thus altering TF binding under patho-physiologic conditions. Furthermore, these findings suggest that PNE-mediated CpG hypomethylation in the AT_2_R gene is partially regulated through the miR-181a signaling pathway. This opens a door for our future studies to see whether and how factors rather than miR-181a regulate PNE-mediated CpG hypomethylation in the AT_2_R gene at the specific ZF-5 binding site.

Numerous studies have shown that both the RAS and TGF-β signaling pathway are critically involved in the pathogenesis of cardiac remodeling, fibrosis and hypertrophy 44-47. Ang II stimulation induces TGF-β1 mRNA and protein expression in cardiomyocytes and cardiac fibroblasts 45. Furthermore, intervention with AT_1_R blockers significantly decreased TGF-β1 levels in hypertrophied 46 and infarcted hearts 47, which suggests that activation of the AT_1_R is the potential molecular linker between Ang II and TGF-β1. These studies also provide evidence that TGF-β1 is a downstream signaling component of the Ang II/ATR in patho-physiologic heart development. TGF-β proteins are synthesized as precursor proteins that are cleaved and reassembled in association with other proteins to form latent complexes. Activation occurs by proteolytic release of TGF-β monomers, which dimerize to form mature TGF-β ligands 48. In the present study, we examined the protein expression status of TGF-β in the offspring heart. Our data indicated that TGF-β latent complexes and TGF-β dimer protein levels were higher in perinatal nicotine-exposed hearts compared to the controls. The up-regulation of TGF-β active dimer expression suggests that the exaggerated TGF-β signaling may be one of the molecular mechanisms underlying nicotine-mediated heart dysfunction and ischemic infarct in rat offspring. Consistent with our result, previous studies have also shown that rats born to prenatal nicotine-treated dams exhibit significantly greater cell width of cardiomyocytes, fewer cardiomyocyte nuclei number associated with higher TGF-β1 expression 49. Over activation of TGF-β is critically involved in cardiac injury 50. Taken together, these studies further suggest that enhanced TGF-β is one of the important biomarkers of PNE-mediated cardiac remodeling, fibrosis, hypertrophy, and ischemic injury.

The Smad family proteins are well known to be one of the major effectors of TGF-β1, which help TGF-β1 signaling to translocate signals from the cell surface directly to the nucleus to regulate target gene transcription. Among these Smad family proteins, Smad2, 3, 4, and 5 are positively regulated by the activation of TGF-β1 signaling. However, Smad6 and 7 serve as a negative feedback loop that regulates TGF-β activity 51. Our present data elucidated that PNE differentially increased Smad2, 3 and 5 protein expressions, but decreased Smad7 protein expression. These data suggest that TGF-β1/Smad2, 3, 5 signaling pathways are up-regulated in response to PNE in the offspring, and the decreased Smad7 could further promote the TGF-β activity.

In the present study, we found that perinatal nicotine-mediated up-regulation of TGF-β expression was rescued by the treatment of LNA-miR-181a. In contrast to our findings, previous studies have been reported that TGF-β can induce miR-181a expression, leading to the increased markers of fibrosis 52. From those findings, it seems unclear whether nicotine-mediated miR-181a is an up-stream or down-stream regulator of TGF-β activity. Interestingly, our current studies have found that inhibition of miR-181a by LNA-miR-181a had no effect on nicotine-mediated Smad2 and 3 over-expression but attenuated the effect of nicotine on Smad5 and Smad 7 expression. These data suggest that PNE-mediated changes of TGF-β/Smad5/7 are regulated by miR-181a signaling, however, the PNE-mediated changes of Smad2/3 are independent of miR-181a signaling. Taken together, from these observations, we could make twofold speculations. First, we speculate that PNE enhances miR-181a expression, which directly activates TGF-β/Smad signaling. Secondly, we also could speculate that PNE will selectively enhance cardiac TGF-β/Smad2/Smad3 expressions, which serve as transcriptional complexes in the nucleus to induce miR-181a expression. The enhanced miR-181a will target and suppress Smad7 expression, which, in turn, diminishes its negative feedback mechanism and consequently indirectly enhances the TGF-β signal pathway (Figure S4).

Autophagy is the most common regulatory mechanism that removes unnecessary dysfunctional components in cells. In pathologic condition, autophagy could serve as an adaptive response to stress, promoting survival of the cell, but in other cases it can also promote cell death and organ dysfunction 20. In the present study, we examined the status of autophagy-related biomarkers including Atg5, Beclin1, p62, LC3B I and LC3B II in the offspring heart tissues. Our data indicated that Atg5, Beclin1, p62 and LC3B-II were overexpressed in PNE hearts as compared to the controls, while LNA-miR-181a treatment eliminated the expression differences of Atg5, Beclin1, p62 and LC3B-II proteins between the saline control and nicotine-exposed groups. These findings suggest that the miR-181a-enhanced autophagic flux may be one of the molecular mechanisms underlying nicotine-mediated heart infraction and dysfunction in rat offspring. In addition, previous studies have suggested that TGF-β could also trigger autophagic flux 53, 54. A previous study demonstrated that inhibition of autophagy reduced TGF-beta2-stimulated RPE cell migration and invasion 53. In another study, overexpression of TGF-beta1 significantly increased protein expression levels of Beclin1 and LC3-II/I, with a concomitant decrease in p62, while TGF-β silencing had the opposite effect 54. This evidence suggests that autophagy signaling acts as a downstream signaling pathway of TGF-β.

In conclusion, our present study provides novel evidence that alteration of miR-181a expression is one of the potential molecular mechanisms underlying PNE-induced development of heart ischemia-sensitive phenotype. Specifically, our data demonstrated PNE induced cardiac miR-181a over-expression and H19 repression, which then down-regulated DNA methylation at ATR promoters with consequent epigenetic up-regulation of ATR expression. The enhanced ATR, in turn, could up-regulate TGF-β/Smads/autophagy signaling pathways leading to increased susceptibility of the heart to I/R-induced injury and cardiac dysfunction in offspring. Of importance, miR-181a antisense rescued perinatal nicotine-mediated changes in ATR/TGF-β/Smads/autophagy signaling and cardiac dysfunction in offspring. Understanding of the epigenetic molecular mechanisms underlying PNE-mediated fetal programming of cardiovascular dysfunction could provide novel therapeutic strategies to prevent, or rescue, the fetal origin of adult cardiovascular disease.

## Limitations and further study

In our present study, we are mainly focusing on the role of miR-181a in PNE-mediated I/R-induce cardiac injury and dysfunction in offspring. However, the molecular mechanisms underlying PNE-mediated miR-181a over-expression in offspring is still unknown. Therefore, in our future studies, we will investigate how PNE programs miR-181a expression in offspring cardiac tissues.

Our present data that the level of lncRNA H19 expression was decreased by LNA-miR-181a treatment, suggests miR-181a could regulate lncRNA H19 expression. However, one of the limitations of present study is that it is unknown whether miR-181a directly or indirectly regulates H19 expression. Therefore, in our future studies we will use Luciferase Reporter Assay to see whether miR-181a directly binds to the H19 gene and affects its promoter activity.

It is well known that epigenetic regulation plays an important role in fetal programming of adult cardiovascular disease. The most common epigenetic mechanisms include DNA methylation, noncoding-RNA (miRNA and lncRNA) regulation, RNA editing, and histone modification. In our present study the data suggests that miRNA/lncRNA regulation and DNA methylation play epigenetic roles in maternal nicotine exposure-induced fetal programming of the cardiac ischemia-sensitive phenotype in postnatal life. In our future studies we will also investigate whether other epigenetic mechanisms such as histone modification are involved.

Because of the technical limitations of Western blot analysis, we cannot load the samples and measure the AT_2_R protein levels of all four groups in the same gel, therefore, our current data cannot tell whether LNA-miR-181a treatment could increase AT_2_R protein levels as it increases mRNA levels (Figure 5B). Similarly, because of the technical limitations of Western blot analysis, from our present data (Figure 6), we cannot make any conclusion about whether LNA-miR-181a treatment has any significant effect on TGFβ/smad 5/7 protein levels under physiological conditions.

Our present findings demonstrated an epigenetic role of miR-181a/DNA methylation in the development of cardiac ischemia-sensitive phenotype in a PNE animal model. Therefore, it will significantly strengthen the clinical implications if we can validate our findings in human studies and translate these findings to humans.

## Supplementary Material

Supplementary figures and tables.

## Abbreviations

PNE: perinatal nicotine exposure
I/R: ischemia/reperfusion
IVSdd/ IVSsd: interventricular septal end diastolic/systolic dimension
LVEDD/ LVESD: left ventricular end diastolic/systolic dimension
LVPWd/LVPWs: left ventricular posterior wall thickness at end diastole/systole
EF: ejection fraction
FS: fractional shortening
SV: stroke volume
LVEDV/ LVESV: left ventricular end-diastolic/ systolic volume
HR: heart rate
LAD: left anterior descending artery
TTC: 2,3,5-triphenyltetrazolium chloride
DNA: deoxyribonucleic acid
Ang II: angiotensin II
TGF-β: transforming growth factor-β
SMAD: small mothers against decapentaplegic
AT1: angiotensin II receptor type 1
AT2: angiotensin II receptor type 2
LC3: microtubule-associated protein light chain 3
Atg5: autophagy related 5

## References

[1] Conklin DJ, Schick S, Blaha MJ, Carll A, DeFilippis A, Ganz P (2019). Cardiovascular injury induced by tobacco products: assessment of risk factors and biomarkers of harm. A Tobacco Centers of Regulatory Science compilation. Am J Physiol Heart Circ Physiol.

[2] Maritz GS, Mutemwa M (2012). Tobacco smoking: patterns, health consequences for adults, and the long-term health of the offspring. Glob J Health Sci.

[3] Blade C, Baselga-Escudero L, Salvado MJ, Arola-Arnal A (2013). MiRNAs, polyphenols, and chronic disease. Mol Nutr Food Res.

[4] Shi J, Bei Y, Kong X, Liu X, Lei Z, Xu T (2017). MiR-17-3p contributes to exercise-induced cardiac growth and protects against myocardial ischemia-reperfusion injury. Theranostics.

[5] Li M, Ding W, Tariq MA, Chang W, Zhang X, Xu W, Hou L (2018). A circular transcript of ncx1 gene mediates ischemic myocardial injury by targeting miR-133a-3p. Theranostics.

[6] Chen P, Pan J, Zhang X, Shi Z, Yang X (2018). The role of microRNA-181a in myocardial fibrosis following myocardial infarction in a rat model. Med Sci Monit.

[7] Li AL, Lv JB, Gao L (2017). MiR-181a mediates Ang II-induced myocardial hypertrophy by mediating autophagy. Eur Rev Med Pharmacol Sci.

[8] Vaskova E, Ikeda G, Tada Y, Wahlquist C, Mercola M, Yang PC (2020). Sacubitril/Valsartan improves cardiac function and decreases myocardial fibrosis *via* downregulation of exosomal miR-181a in a rodent chronic myocardial infarction model. J Am Heart Assoc.

[9] Zhu J, Yao K, Wang Q (2016). Circulating miR-181a as a potential novel biomarker for diagnosis of acute myocardial infarction. Cell Physiol Biochem.

[10] Liu B, Hu X, Li Y, Ke J, Dasgupta C, Huang X (2019). Epigenetic down-regulation of BKCa channel by miR-181a contributes to the fetal and neonatal nicotine-mediated exaggerated coronary vascular tone in adult life. Int J Cardiol.

[11] Lawrence J, Xiao D, Xue Q, Rejali M, Yang S, Zhang L (2008). Prenatal nicotine exposure increases heart susceptibility to ischemia/reperfusion injury in adult offspring. J Pharmacol Exp Ther.

[12] Lorenzen JM, Thum T (2016). Long noncoding RNAs in kidney and cardiovascular diseases. Nat Rev Nephrol.

[13] Jiang XY, Feng YL, Ye LT, Li XH, Feng J, Zhang MZ (2017). Inhibition of Gata4 and Tbx5 by nicotine-mediated DNA methylation in myocardial differentiation. Stem Cell Reports.

[14] Bujak M, Frangogiannis NG (2007). The role of TGF-β signaling in myocardial infarction and cardiac remodeling. Cardiovasc Res.

[15] Yildiz M, Oktay AA, Stewart MH, Milani RV, Ventura HO, Lavie CJ (2020). Left ventricular hypertrophy and hypertension. Prog Cardiovasc Dis.

[16] Mehta PK, Griendling KK (2007). Angiotensin II cell signaling: physiological and pathological effects in the cardiovascular system. Am J Physiol Cell Physiol.

[17] Xie F, Zhang Z, van Dam H, Zhang L, Zhou F (2014). Regulation of TGF-beta superfamily signaling by SMAD mono-ubiquitination. Cells.

[18] Qi HP, Wang Y, Zhang QH, Guo J, Li L, Cao YG (2015). Activation of peroxisome proliferator-activated receptor gamma (PPARgamma) through NF-kappaB/Brg1 and TGF-beta1 pathways attenuates cardiac remodeling in pressure-overloaded rat hearts. Cell Physiol Biochem.

[19] Marcin Dobaczewski, Wei Chen, Nikolaos G Frangogiannis (2011). Transforming growth factor (TGF)-β signaling in cardiac remodeling. J Mol Cell Cardiol.

[20] Tian J, Popal MS, Zhao Y, Liu Y, Chen K, Liu Y (2019). Interplay between exosomes and autophagy in cardiovascular diseases: novel promising target for diagnostic and therapeutic application. Aging Dis.

[21] Fewell JE, Smith FG, Ng VK (2001). Threshold levels of maternal nicotine impairing protective responses of newborn rats to intermittent hypoxia. J Appl Physiol. (1985).

[22] Chen Z, Gong L, Zhang P, Li Y, Liu B, Zhang L (2019). Epigenetic down-regulation of sirt 1 *via* DNA methylation and oxidative stress signaling contributes to the gestational diabetes mellitus-induced fetal programming of heart ischemia-sensitive phenotype in late life. Int J Biol Sci.

[23] Zhang P, Lv J, Li Y, Zhang L, Xiao D (2017). Neonatal lipopolysaccharide exposure gender-dependently increases heart susceptibility to ischemia/reperfusion injury in male rats. Int J Med Sci.

[24] Garchow BG, Bartulos Encinas O, Leung YT, Tsao PY, Eisenberg RA, Caricchio R (2011). Silencing of microRNA-21 *in vivo* ameliorates autoimmune splenomegaly in lupus mice. EMBO Mol Med.

[25] Goedeke L, Rotllan N, Canfran-Duque A, Aranda JF, Ramirez CM, Araldi E (2015). MicroRNA-148a regulates LDL receptor and ABCA1 expression to control circulating lipoprotein levels. Nat Med.

[26] Zhou B, Li C, Qi W, Zhang Y, Zhang F, Wu JX (2012). Downregulation of miR-181a upregulates sirtuin-1 (SIRT1) and improves hepatic insulin sensitivity. Diabetologia.

[27] Gopalakrishnan K, More AS, Hankins GD, Nanovskaya TN, Kumar S (2017). Postnatal cardiovascular consequences in the offspring of pregnant rats exposed to smoking and smoking cessation pharmacotherapies. Reprod Sci.

[28] Vivekanandarajah A, Waters KA, Machaalani R (2019). Cigarette smoke exposure effects on the brainstem expression of nicotinic acetylcholine receptors (nAChRs), and on cardiac, respiratory and sleep physiologies. Respir Physiol Neurobiol.

[29] Xiao D, Wang L, Huang X, Li Y, Dasgupta C, Zhang L (2016). Protective effect of antenatal antioxidant on nicotine-induced heart ischemia-sensitive phenotype in rat offspring. Plos One.

[30] Ke J, Dong N, Wang L, Li Y, Dasgupta C, Zhang L (2017). Role of DNA methylation in perinatal nicotine-induced development of heart ischemia-sensitive phenotype in rat offspring. Oncotarget.

[31] Xiao D, Huang X, Lawrence J, Yang S, Zhang L (2007). Fetal and neonatal nicotine exposure differentially regulates vascular contractility in adult male and female offspring. J Pharmacol Exp Ther.

[32] Xiao D, Xu Z, Huang X, Longo LD, Yang S, Zhang L (2008). Prenatal gender-related nicotine exposure increases blood pressure response to angiotensin II in adult offspring. Hypertension.

[33] Xiao D, Huang X, Yang S, Zhang L (2013). Estrogen normalizes perinatal nicotine- induced hypertensive responses in adult female rat offspring. Hypertension.

[34] Boon RA, Dimmeler S (2015). MicroRNAs in myocardial infarction. Nat Rev Cardiol.

[35] Li L, Wang Q, Yuan Z, Chen A, Liu Z, Li H (2018). Long non-coding RNA H19 contributes to hypoxia-induced CPC injury by suppressing Sirt1 through miR-200a-3p. Acta Biochim Biophys Sin (Shanghai).

[36] Liu L, An X, Li Z, Song Y, Li L, Zuo S (2016). The H19 long noncoding RNA is a novel negative regulator of cardiomyocyte hypertrophy. Cardiovasc Res.

[37] Kapoor D, Jones TH (2005). Smoking and hormones in health and endocrine disorders. Eur J Endocrinol.

[38] Laustiola KE, Lassila R, Nurmi AK (1988). Enhanced activation of the renin-angiotensin-aldosterone system in chronic cigarette smokers: a study of monozygotic twin pairs discordant for smoking. Clin Pharmacol Ther.

[39] Nakayama M, Yan X, Price RL, Borg TK, Ito K, Sanbe A (2005). Chronic ventricular myocyte-specific overexpression of angiotensin II type 2 receptor results in intrinsic myocyte contractile dysfunction. Am J Physiol Heart Circ Physiol.

[40] Oakes JM, Fuchs RM, Gardner JD, Lazartigues E, Yue X (2018). Nicotine and the renin-angiotensin system. Am J Physiol Regul Integr Comp Physiol.

[41] Colombo ES, Davis J, Makvandi M, Aragon M, Lucas SN, Paffett ML (2013). Effects of nicotine on cardiovascular remodeling in a mouse model of systemic hypertension. Cardiovasc Toxicol.

[42] Narayanaswami V, Somkuwar SS, Horton DB, Cassis LA, Dwoskin LP (2013). Angiotensin AT1 and AT2 receptor antagonists modulate nicotine-evoked [(3)H]dopamine and [(3)H]norepinephrine release. Biochem Pharmacol.

[43] Xiao D, Dasgupta C, Li Y, Huang X, Zhang L (2014). Perinatal nicotine exposure increases angiotensin II receptor-mediated vascular contractility in adult offspring. Plos One.

[44] Rosenkranz S (2004). TGF-beta1 and angiotensin networking in cardiac remodeling. Cardiovasc Res.

[45] Gray MO, Long CS, Kalinyak JE, Li HT, Karliner JS (1998). Angiotensin II stimulates cardiac myocyte hypertrophy *via* paracrine release of TGF-beta 1 and endothelin-1 from fibroblasts. Cardiovasc Res.

[46] Kim S, Ohta K, Hamaguchi A, Yukimura T, Miura K, Iwao H (1996). Effects of an AT1 receptor antagonist, an ACE inhibitor and a calcium channel antagonist on cardiac gene expressions in hypertensive rats. Br J Pharmacol.

[47] Hao J, Wang B, Jones SC, Jassal DS, Dixon IM (2000). Interaction between angiotensin II and Smad proteins in fibroblasts in failing heart and *in vitro*. Am J Physiol Heart Circ Physiol.

[48] Kingsley DM (1994). The TGF-beta superfamily: new members, new receptors, and new genetic tests of function in different organisms. Genes Dev.

[49] Chou HC, Chen CM (2014). Maternal nicotine exposure during gestation and lactation induces cardiac remodeling in rat offspring. Reprod Toxicol.

[50] Sakata Y, Chancey AL, Divakaran VG, Sekiguchi K, Sivasubramanian N, Mann DL (2008). Transforming growth factor-beta receptor antagonism attenuates myocardial fibrosis in mice with cardiac-restricted overexpression of tumor necrosis factor. Basic Res Cardiol.

[51] Hayashi H, Abdollah S, Qiu Y, Cai J, Xu YY, Grinnell BW (1997). The MAD-related protein Smad7 associates with the TGFbeta receptor and functions as an antagonist of TGFbeta signaling. Cell.

[52] Gupta P, Sata TN, Yadav AK, Mishra A, Vats N, Hossain MM (2019). TGF-beta induces liver fibrosis *via* miRNA-181a-mediated down regulation of augmenter of liver regeneration in hepatic stellate cells. Plos One.

[53] Wu J, Chen X, Liu X, Huang S, He C, Chen B (2018). Autophagy regulates TGF-beta2-induced epithelial-mesenchymal transition in human retinal pigment epithelium cells. Mol Med Rep.

[54] Zhao Y, Li Y, Gao Y, Yuan M, Manthari RK, Wang J (2018). TGF-beta1 acts as mediator in fluoride-induced autophagy in the mouse osteoblast cells. Food Chem Toxicol.

