# Supplementary material for: Reprogramming of miR-181a/DNA methylation patterns contribute to the maternal nicotine exposure-induced fetal programming of cardiac ischemia-sensitive phenotype in postnatal life
Source: Theranostics. 2020 Sep 26;10(25):11820–36. doi: 10.7150/thno.48297 (PMC7546014; doi:10.7150/thno.48297)
Supplement: Supplementary file 1 — Supplementary figures and tables. [file thnov10p11820s1.pdf]

Supplemental Data

Reprogramming of miR-181a/DNA methylation patterns contribute to the maternal nicotine exposure-induced fetal programming of cardiac ischemia-sensitive phenotype in postnatal life

Jie Jian[1,2], Peng Zhang[1], Yong Li[1], Bailin Liu[1], Yanyan Zhang[1], Lubo Zhang[1], Xuesi M Shao[3], Jian Zhuang[2], and Daliao Xiao[1]

[1] Lawrence D. Longo, MD Center for Perinatal Biology, Department of Basic Sciences, Loma Linda University School of Medicine, Loma Linda, California, USA

[2] Department of Cardiac Surgery, Guangdong Cardiovascular Institute, Guangdong Provincial People's Hospital, Guangdong Academy of Medical Sciences, Guangzhou, China

[3] Department of Neurobiology, David Geffen School of Medicine at UCLA, University of California at Los Angeles, Los Angeles, California, USA

a) **Corresponding authors:**

Daliao Xiao, PhD
Center for Perinatal Biology
Department of Basic Sciences
Loma Linda University School of Medicine
Loma Linda, CA 92350
Tel: 909-558-4325, Fax: 909-558-4029
E-mail: Dxiao@llu.edu

Jian Zhuang, MD
Department of Cardiac Surgery
Guangdong Cardiovascular Institute
Guangdong Provincial People's Hospital
Guangdong Academy of Medical Sciences
Guangzhou, China 510000
Tel: 86 020 83827812
E-mail: zhuangj0130@163.com

	Baseline		1 Day after I/R		7 Days after I/R	
	Saline (n=10)	Nicotine (n=10)	Saline (n=5)	Nicotine (n=6)	Saline (n=4)	Nicotine (n=4)
IVSdd(cm)	0.171 ± 0.008	0.155 ± 0.007	0.180 ± 0.004	0.165 ± 0.009	0.155 ± 0.009	0.158 ± 0.008
IVSsd (cm)	0.261 ± 0.011	0.248 ± 0.007	0.185 ± 0.009	0.176 ± 0.014	0.165 ± 0.009	0.218 ± 0.027
LVEDD(cm)	0.589 ± 0.021	0.573 ± 0.014	0.620 ± 0.042	0.607 ± 0.027	0.630 ± 0.015	0.663 ± 0.011
LVESD(cm)	0.321 ± 0.020	0.304 ± 0.018	0.416 ± 0.027	0.423 ± 0.023	0.433 ± 0.019	0.460 ± 0.011
LVPWd(cm)	0.221 ± 0.018	0.215 ± 0.007	0.210 ± 0.020	0.187 ± 0.009	0.178 ± 0.015	0.205 ± 0.013
LVPWs(cm)	0.276 ± 0.007	0.299 ± 0.010	0.272 ± 0.019	0.248 ± 0.012	0.270 ± 0.021	0.275 ± 0.021
LVEDV(ml)	0.490 ± 0.053	0.445 ± 0.032	0.610 ± 0.094	0.537 ± 0.065	0.580 ± 0.036	0.700 ± 0.012*
LVESV(ml)	0.089 ± 0.016	0.080 ± 0.013	0.214 ± 0.037	0.193 ± 0.030	0.200 ± 0.025	0.248 ± 0.015
EF(%)	81.89 ± 2.314	82.28 ± 2.641	65.61 ± 1.724	64.41 ± 2.110	65.79 ± 2.281	64.55 ± 2.392
FS(%)	45.83 ± 2.599	46.51 ± 2.873	33.30 ± 1.637	30.77 ± 1.484	31.72 ± 1.542	31.94 ± 1.600
SV(ml)	0.401 ± 0.042	0.364 ± 0.028	0.366 ± 0.055	0.342 ± 0.036	0.380 ± 0.015	0.478 ± 0.022*
HR(bpm)	385.0 ± 12.79	362.8 ± 19.07	444.6 ± 8.07	400.0 ± 20.61	389.5 ± 22.02	350.0 ± 23.51

Table S1. Effect of PNE on LV parameters determined by echocardiography in female offspring on baseline, 1 day before I/R, 1 day after I/R, 7 days after I/R

Note: IVSdd/ IVSsd, interventricular septal end diastolic/systolic dimension; LVEDD/ LVESD, left ventricular end diastolic/systolic dimension; LVPWd/ LVPWs, left ventricular posterior wall thickness at end diastole/systole; EF, ejection fraction; FS, fractional shortening; SV, stroke volume; LVEDV/ LVESV, left ventricular end-diastolic/ systolic volume; HR, heart rate; *P < 0.05 versus saline control group, as determined by Student's t-test.

	Baseline		1 Day before I/R		1 Day after I/R		7 Days after I/R	
	Saline (n=10)	Nicotine (n=10)	Saline (n=10)	Nicotine (n=10)	Saline (n=9)	Nicotine (n=9)	Saline (n=5)	Nicotine (n=3)
IVSdd(cm)	0.169 ± 0.003	0.151 ± 0.004*	0.184 ± 0.005	0.139 ± 0.003*	0.178 ± 0.005	0.160 ± 0.006*	0.183 ± 0.012	0.142 ± 0.002*
IVSsd (cm)	0.302 ± 0.006	0.248 ± 0.008*	0.309 ± 0.012	0.244 ± 0.008*	0.250 ± 0.010	0.205 ± 0.013*	0.259 ± 0.017	0.195 ± 0.023
LVEDD(cm)	0.690 ± 0.022	0.670 ± 0.024	0.704 ± 0.015	0.695 ± 0.025	0.744 ± 0.013	0.725 ± 0.018	0.739 ± 0.016	0.761 ± 0.042
LVESD(cm)	0.361 ± 0.017	0.386 ± 0.019	0.361 ± 0.018	0.403 ± 0.017	0.478 ± 0.010	0.513 ± 0.005*	0.483 ± 0.011	0.544 ± 0.015*
LVPWd(cm)	0.248 ± 0.012	0.224 ± 0.012	0.250 ± 0.008	0.232 ± 0.012	0.241 ± 0.015	0.232 ± 0.012	0.238 ± 0.008	0.216 ± 0.006
LVPWs(cm)	0.320 ± 0.010	0.284 ± 0.006*	0.329 ± 0.011	0.291 ± 0.010*	0.316 ± 0.011	0.276 ± 0.013*	0.297 ± 0.009	0.264 ± 0.013
LVEDV(ml)	0.764 ± 0.062	0.705 ± 0.069	0.802 ± 0.046	0.783 ± 0.071	0.924 ± 0.041	0.864 ± 0.058	0.916 ± 0.054	1.005 ± 0.149
LVESV(ml)	0.126 ± 0.015	0.154 ± 0.021	0.128 ± 0.018	0.170 ± 0.018	0.265 ± 0.014	0.326 ± 0.009*	0.280 ± 0.019	0.390 ± 0.029*
EF(%)	84.53 ± 0.743	79.02 ± 1.047*	84.52 ± 1.562	78.27 ± 1.219*	70.80 ± 1.612	61.27 ± 2.086*	69.79 ± 0.619	59.85 ± 2.901*
FS(%)	48.10 ± 0.985	42.06 ± 0.967*	49.01 ± 1.852	42.05 ± 1.115*	35.75 ± 1.246	28.85 ± 1.435*	34.82 ± 0.454	28.10 ± 1.950*
SV(ml)	0.687 ± 0.028	0.587 ± 0.027*	0.675 ± 0.033	0.576 ± 0.031*	0.583 ± 0.023	0.472 ± 0.029*	0.572 ± 0.018	0.426 ± 0.055*
HR(bpm)	371.2 ± 13.05	352.7 ± 8.40	342.1 ± 11.66	342.1 ± 8.81	374.3 ± 10.15	377.3 ± 7.62	359.4 ± 16.48	374.1 ± 28.97

Table S2. Effect of PNE on LV parameters determined by echocardiography in male offspring on baseline, 1 day before I/R, 1 day after I/R, 7 days after I/R

Note: IVSdd/ IVSsd, interventricular septal end diastolic/systolic dimension; LVEDD/ LVESD, left ventricular end diastolic/systolic dimension; LVPWd/ LVPWs, left ventricular posterior wall thickness at end diastole/systole; EF, ejection fraction; FS, fractional shortening; SV, stroke volume; LVEDV/ LVESV, left ventricular end-diastolic/ systolic volume; HR, heart rate; *$P < 0.05$ versus saline control group, as determined by Student's t-test.

	Baseline		1 Day before I/R		1 Day after I/R		7 Days after I/R	
	Saline (n=10)	Nicotine (n=10)	Saline (n=9)	Nicotine (n=7)	Saline (n=9)	Nicotine (n=9)	Saline (n=4)	Nicotine (n=5)
IVSdd(cm)	0.167 ± 0.006	0.139 ± 0.007*	0.172 ± 0.004	0.153 ± 0.006*	0.170 ± 0.005	0.144 ± 0.003*	0.171 ± 0.003	0.149 ± 0.006*
IVSsd (cm)	0.290 ± 0.007	0.231 ± 0.008*	0.309 ± 0.008	0.272 ± 0.011*	0.223 ± 0.011	0.219 ± 0.009	0.255 ± 0.010	0.228 ± 0.009
LVEDD(cm)	0.652 ± 0.019	0.640 ± 0.023	0.669 ± 0.011	0.652 ± 0.021	0.712 ± 0.016	0.715 ± 0.015	0.775 ± 0.034	0.751 ± 0.028
LVESD(cm)	0.332 ± 0.014	0.361 ± 0.014	0.356 ± 0.013	0.342 ± 0.015	0.464 ± 0.014	0.479 ± 0.017	0.483 ± 0.011	0.509 ± 0.023
LVPWd(cm)	0.233 ± 0.009	0.208 ± 0.008	0.220 ± 0.006	0.215 ± 0.009	0.255 ± 0.014	0.246 ± 0.013	0.221 ± 0.010	0.210 ± 0.015
LVPWs(cm)	0.313 ± 0.010	0.260 ± 0.009*	0.344 ± 0.007	0.315 ± 0.016	0.288 ± 0.016	0.258 ± 0.015	0.279 ± 0.029	0.243 ± 0.011
LVEDV(ml)	0.651 ± 0.053	0.620 ± 0.056	0.709 ± 0.035	0.621 ± 0.057	0.819 ± 0.051	0.832 ± 0.049	1.040 ± 0.117	0.966 ± 0.099
LVESV(ml)	0.100 ± 0.012	0.122 ± 0.012	0.119 ± 0.012	0.136 ± 0.039	0.248 ± 0.021	0.279 ± 0.026	0.309 ± 0.038	0.325 ± 0.042
EF(%)	85.24 ± 0.730	79.86 ± 1.682*	83.60 ± 0.996	82.44 ± 1.792	69.64 ± 1.293	67.14 ± 1.565	69.16 ± 1.766	66.34 ± 1.572
FS(%)	49.25 ± 0.850	43.55 ± 1.448*	47.60 ± 1.114	46.58 ± 1.929	34.76 ± 0.948	32.97 ± 1.112	34.45 ± 1.260	32.47 ± 1.158
SV(ml)	0.721 ± 0.016	0.679 ± 0.011*	0.570 ± 0.038	0.516 ± 0.049	0.513 ± 0.024	0.459 ± 0.017	0.503 ± 0.040	0.446 ± 0.019
HR(bpm)	383.6 ± 10.18	362.4 ± 12.49	351.5 ± 19.60	365.8 ± 14.01	376.4 ± 15.10	393.5 ± 17.28	410.9 ± 19.79	394.2 ± 10.88

Table S3. Effect of PNE on LV parameters determined by echocardiography in male offspring with LNA-miR-181a treatment on baseline, 1 day before I/R, 1 day after I/R, 7 days after I/R

Note: IVSdd/ IVSsd, interventricular septal end diastolic/systolic dimension; LVEDD/ LVESD, left ventricular end diastolic/systolic dimension; LVPWd/ LVPWs, left ventricular posterior wall thickness at end diastole/systole; EF, ejection fraction; FS, fractional shortening; SV, stroke volume; LVEDV/ LVESV, left ventricular end-diastolic/ systolic volume; HR, heart rate; *P < 0.05 versus saline control group, as determined by Student's t-test.

	Baseline		1 Day before I/R		1 Day after I/R		7 Days after I/R	
	**Saline** (n=11)	**Scramble** (n=11)	**Saline** (n=11)	**Scramble** (n=11)	**Saline** (n=5)	**Scramble** (n=5)	**Saline** (n=5)	**Scramble** (n=5)
IVSdd(cm)	0.135 ± 0.006	0.133 ± 0.003	0.137 ± 0.008	0.150 ± 0.005	0.146 ± 0.013	0.138 ± 0.009	0.134 ± 0.012	0.130 ± 0.009
IVSsd (cm)	0.255 ± 0.012	0.255 ± 0.009	0.278 ± 0.012	0.279 ± 0.008	0.212 ± 0.027	0.206 ± 0.014	0.216 ± 0.025	0.194 ± 0.014
LVEDD(cm)	0.624 ± 0.024	0.638 ± 0.017	0.689 ± 0.015	0.711 ± 0.013	0.698 ± 0.025	0.730 ± 0.021	0.820 ± 0.034	0.836 ± 0.036
LVESD(cm)	0.301 ± 0.018	0.313 ± 0.012	0.335 ± 0.013	0.353 ± 0.013	0.438 ± 0.023	0.470 ± 0.023	0.540 ± 0.025	0.560 ± 0.029
LVPWd(cm)	0.215 ± 0.015	0.210 ± 0.007	0.195 ± 0.008	0.214 ± 0.011	0.210 ± 0.015	0.194 ± 0.007	0.206 ± 0.009	0.195 ± 0.021
LVPWs(cm)	0.310 ± 0.016	0.318 ± 0.016	0.307 ± 0.009	0.329 ± 0.009	0.293 ± 0.029	0.276 ± 0.012	0.326 ± 0.013	0.308 ± 0.028
LVEDV(ml)	0.580 ± 0.062	0.609 ± 0.047	0.749 ± 0.045	0.817 ± 0.042	0.690 ± 0.096	0.883 ± 0.072	1.218 ± 0.122	1.292 ± 0.137
LVESV(ml)	0.082 ± 0.012	0.082 ± 0.010	0.102 ± 0.010	0.115 ± 0.013	0.235 ± 0.041	0.285 ± 0.033	0.384 ± 0.048	0.428 ± 0.059
EF(%)	86.36 ± 1.074	86.67 ± 0.950	86.60 ± 0.986	86.24 ± 1.100	70.33 ± 1.563	67.77 ± 2.541	68.64 ± 1.676	67.10 ± 2.002
FS(%)	50.74 ± 1.371	51.08 ± 1.232	51.17 ± 1.324	50.79 ± 1.376	35.08 ± 1.196	33.48 ± 1.871	34.17 ± 1.268	33.09 ± 1.409
SV(ml)	0.498 ± 0.051	0.527 ± 0.041	0.648 ± 0.038	0.703 ± 0.033	0.530 ± 0.054	0.630 ± 0.056	0.838 ± 0.082	0.862 ± 0.089
HR(bpm)	375.3 ± 15.82	405.8 ± 10.61	380.9 ± 15.20	379.9 ± 9.15	379.0 ± 16.98	381.7 ± 21.79	355.0 ± 31.14	356.7 ± 16.66

Table S4. Effect of scrambled LNA-miR-181a on LV parameters determined by echocardiography in ~ 3-month-old male rats on baseline, 1 day before I/R, 1 day after I/R, 7 days after I/R

Note: IVSdd/ IVSsd, interventricular septal end diastolic/systolic dimension; LVEDD/ LVESD, left ventricular end diastolic/systolic dimension; LVPWd/ LVPWs, left ventricular posterior wall thickness at end diastole/systole; EF, ejection fraction; FS, fractional shortening; SV, stroke volume; LVEDV/ LVESV, left ventricular end-diastolic/ systolic volume; HR, heart rate; **P* < 0.05 versus saline control group, as determined by Student’s t-test.

Figure S1. Effects of PNE on ischemia-reperfusion-induced heart infarct size in female offspring rats. Rats were administered with either saline or nicotine from gestation day 4 until postnatal day 10. 3-month-old offspring rats from each group were subjected to 45 min of heart ischemia followed by reperfusion. For the infarction size, the hearts of rats were isolated 24 h after I/R and their infarct sizes in each rat group were determined with 2% TTC staining (A), (B) showing percent of left ventricle infarct size (infarct area/ left ventricle area x100%) in each offspring group (n=4 animals/group). *$P < 0.05$ versus saline control group, as determined by Student's t-test.

Figure S2. Effects of PNE on heart function in female offspring rats. Rats were administered with either saline or nicotine from gestation day 4 until postnatal day 10. 3-month-old rats from each group were subjected to 45 min of heart ischemia followed by reperfusion. The representative echocardiographic images were obtained from different time periods including baseline (A), 1 day before I/R, 1 day after I/R, and 7 days after I/R. (B) left ventricular end-diastolic dimension (LVEDD), (C) left ventricular end-systolic dimension (LVESD), (D) percentage of ejection fraction (EF%), (E) percentage of fractional shortening (FS%) (n=4~10 animals/group). *$P < 0.05$ versus saline control group, as determined by Student's t-test.

Figure S3. Effects of scrambled LNA-miR-181a on heart function in male rats. Rats were administered with either saline solution or scrambled LNA-miR-181a (negative control) 10 days before I/R. 3-month-old rats from each group were subjected to 45 min of heart ischemia followed by reperfusion. The representative echocardiographic images were obtained from different time periods including baseline (A), 1 day before I/R, 1 day after I/R, and 7 days after I/R. (B) left ventricular end-diastolic dimension (LVEDD), (C) left ventricular end-systolic dimension (LVESD), (D) percentage of ejection fraction (EF%), (E) percentage of fractional shortening (FS%) (n=5~11 animals/group). *$P < 0.05$ versus saline control group, as determined by

Student's t-test.

Figure S4. Effects of scrambled LNA-miR-181a on miR-181a levels in male rat and effect of PNE on miR-181b/c levels in offspring rats. The total RNA samples were isolated from left ventricle tissues of either normal male rats or the male offspring rats from each group. Cardiac miR-181a, 181b, 181c levels were determined by qRT-PCR analysis as described in the Material and Methods. (A) miR-181a levels in the saline solution group and the scrambled LNA-miR-181a-treated rats (n=6 animals/group), (B) miR-181b levels in each group of offspring (n=6 animals/group), (C) miR-181c levels in each group of offspring (n=5-6 animals/group). Data are means ± SEM. The expression levels of miR-181 family are normalized by SNOTD61. *P < 0.05 versus saline control group, as determined by Student's t-test.

Figure S5. A diagram showing the TGF-β/Smad signaling pathway interaction with miR-181a in response to perinatal nicotine exposure (PNE). PNE selectively enhances cardiac TGF-β/Smad2/Smad3 expressions, which serve as transcriptional complexes in the nucleus to induce miR-181a expression. The enhanced miR-181a will target and suppress Smad7 expression, which, in turn, diminishes its negative feedback mechanism and consequently indirectly enhances TGF-β signal pathway.

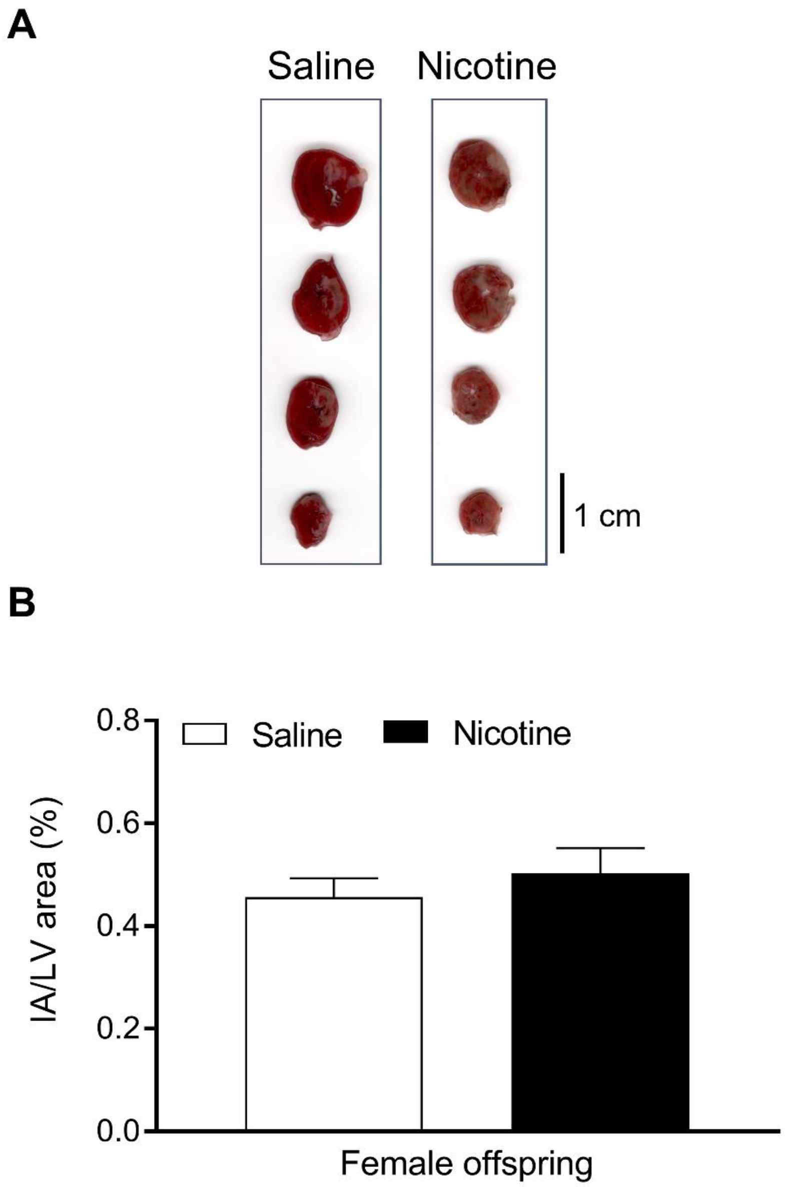
A
Saline
Nicotine
1 cm
B
0.8
0.6
0.4
0.2
0.0
Saline
Nicotine
IA/LV area (%)
Female offspring

Figure S1

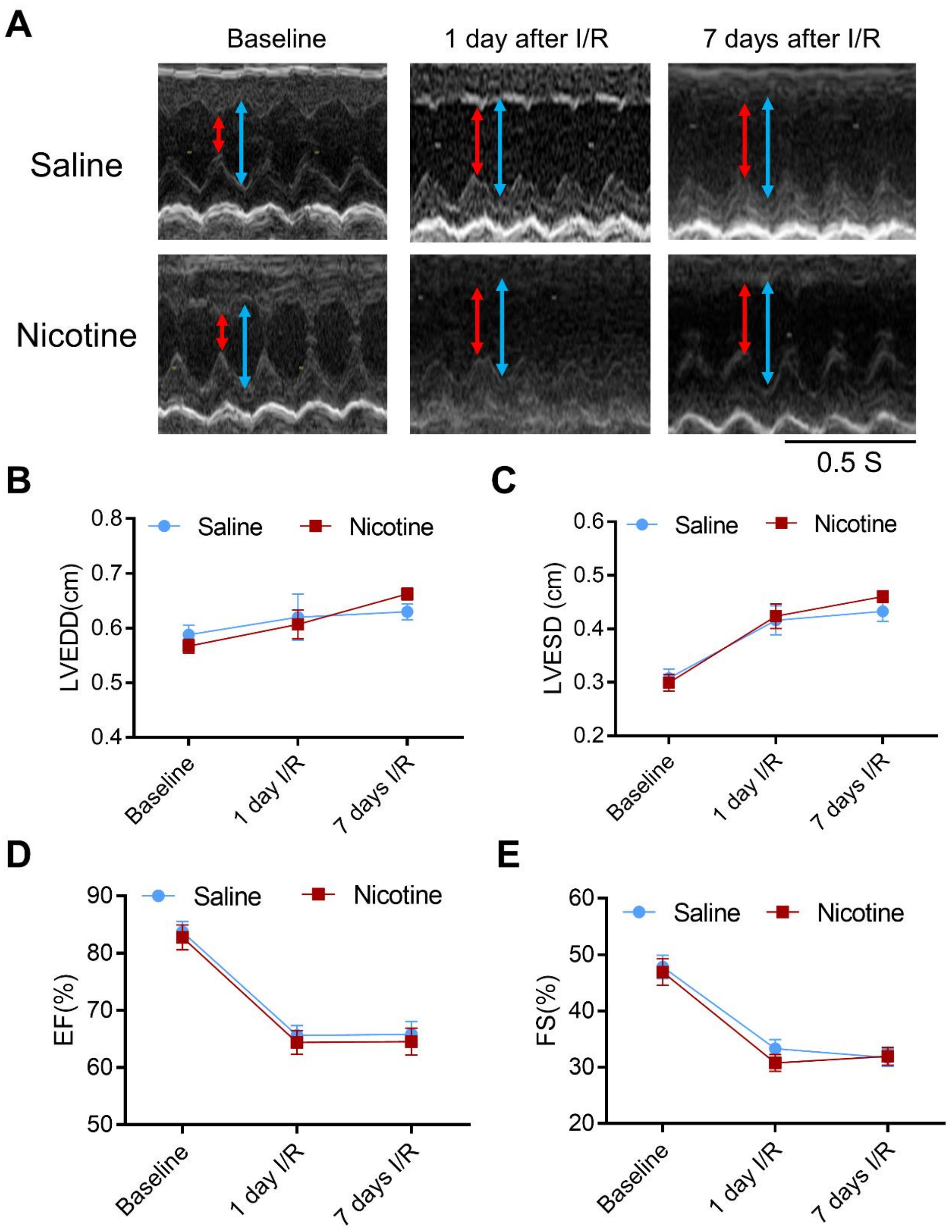
A
Baseline
1 day after I/R
7 days after I/R
Saline
Nicotine
0.5 S
B
0.8
0.7
0.6
0.5
0.4
LVEDD(cm)
Saline
Nicotine
Baseline
1 day I/R
7 days I/R
C
0.6
0.5
0.4
0.3
0.2
LVESD (cm)
Saline
Nicotine
Baseline
1 day I/R
7 days I/R
D
90
80
70
60
50
EF(%)
Saline
Nicotine
Baseline
1 day I/R
7 days I/R
E
60
50
40
30
20
FS(%)
Saline
Nicotine
Baseline
1 day I/R
7 days I/R

Figure S2

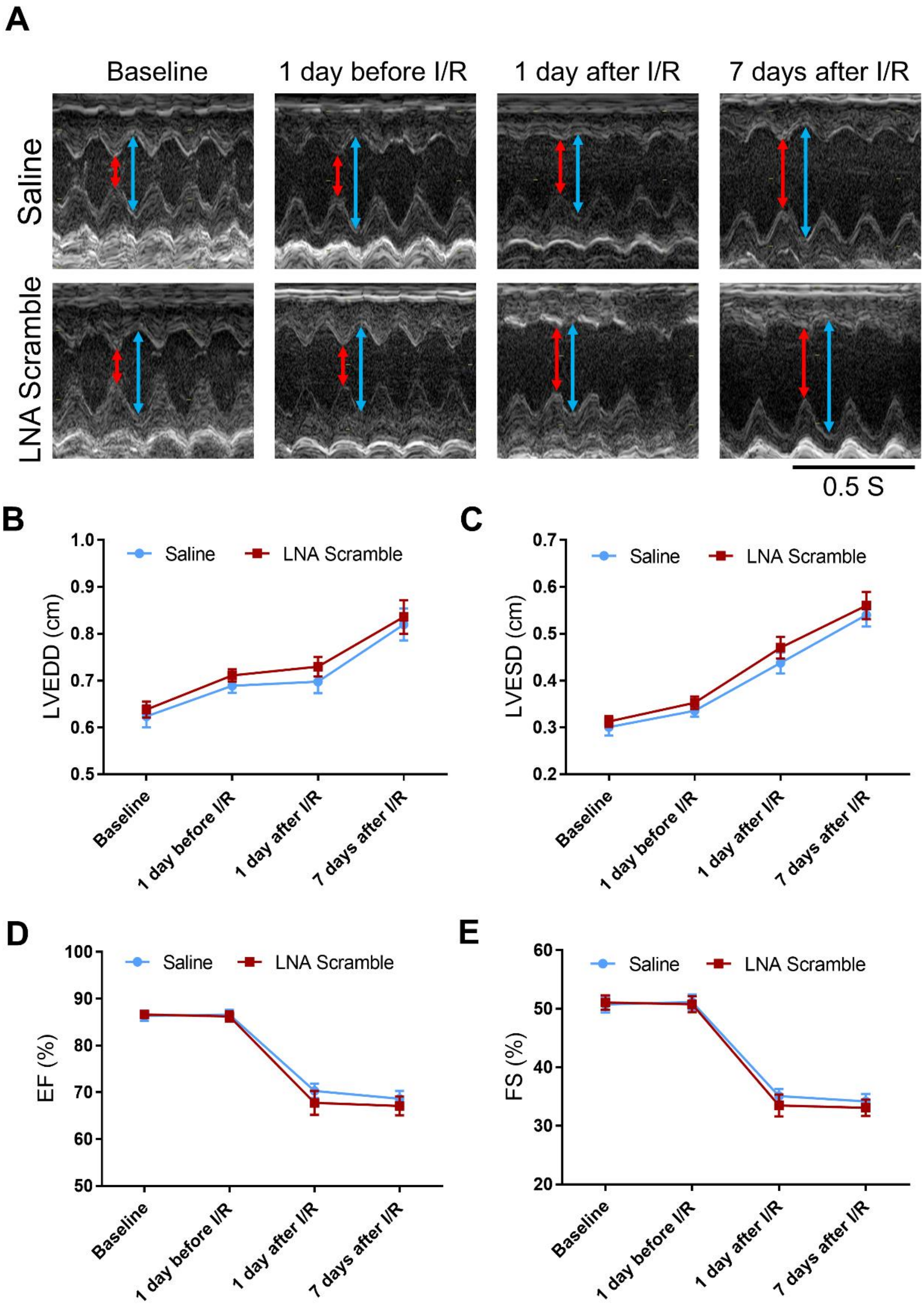
A
Baseline
1 day before I/R
1 day after I/R
7 days after I/R
Saline
LNA Scramble
0.5 S
B
LVEDD (cm)
Saline
LNA Scramble
C
LVESD (cm)
D
EF (%)
E
FS (%)
Baseline
1 day before I/R
1 day after I/R
7 days after I/R

Figure S3

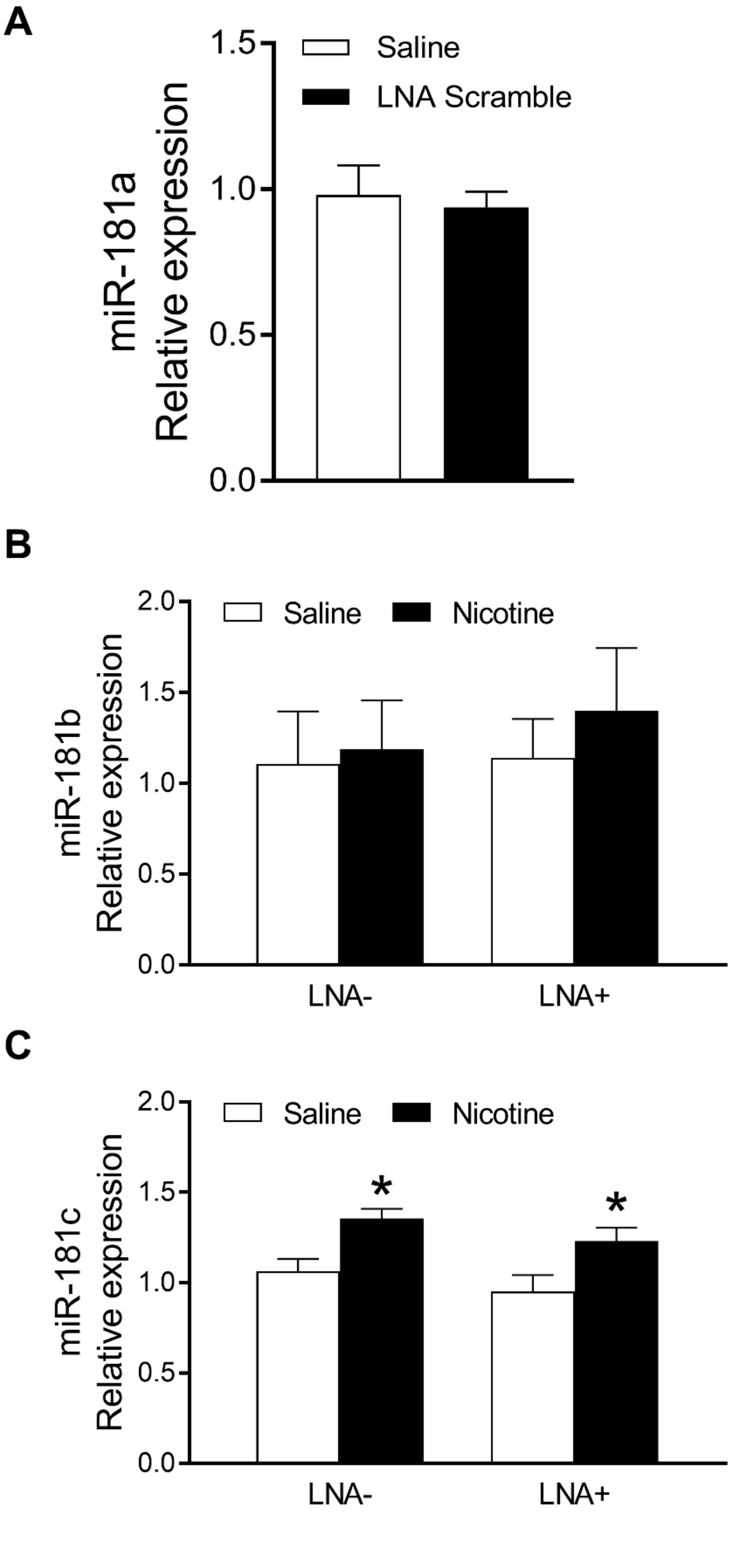
A
1.5
1.0
0.5
0.0
miR-181a
Relative expression
Saline
LNA Scramble
B
2.0
1.5
1.0
0.5
0.0
miR-181b
Relative expression
Saline
Nicotine
LNA-
LNA+
C
2.0
1.5
1.0
0.5
0.0
miR-181c
Relative expression
Saline
Nicotine
*
*
LNA-
LNA+

Figure S4

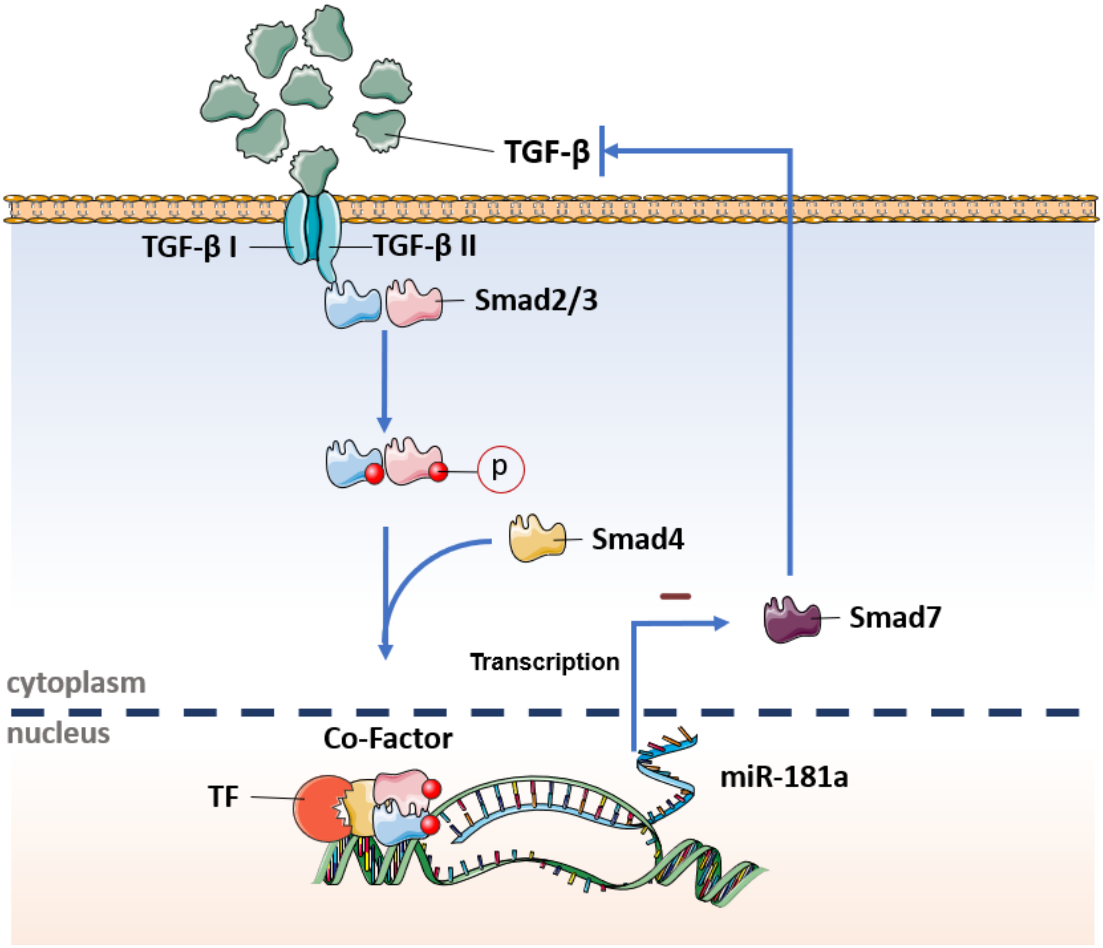
TGF-β
TGF-β I
TGF-β II
Smad2/3
p
Smad4
Smad7
Transcription
cytoplasm
nucleus
Co-Factor
TF
miR-181a

Figure S5
